# Structural basis for activation and potentiation in a human α5β3 GABA_A_ receptor

**DOI:** 10.1038/s41467-026-74279-3

**Published:** 2026-06-15

**Authors:** John Cowgill, Chen Fan, Jan Steyaert, Rebecca J. Howard, Erik Lindahl

**Affiliations:** 1 Department of Biochemistry and Biophysics, SciLifeLab, Stockholm University, Solna, Sweden; 2 Department of Applied Physics, SciLifeLab, KTH Royal Institute of Technology, Solna, Sweden; 3 Department of Pharmacology and Chemical Biology, Shanghai Jiao Tong University School of Medicine, Shanghai, China; 4 VIB-VUB Center for Structural Biology, VIB, Brussels, Belgium; 5 Structural Biology Brussels, Vrije Universiteit Brussel, Brussels, Belgium; 6 Department of Physics, Chemistry and Biology, Linköping University, Linköping, Sweden; 7 Department of Chemistry, University of Illinois at Urbana-Champaign, Urbana, IL USA

**Keywords:** Cryoelectron microscopy, Ligand-gated ion channels, Permeation and transport

## Abstract

Anesthetics and anticonvulsants act, in part, through diverse populations of type-A ɣ-aminobutyric acid receptors (GABA_A_Rs) formed from a pool of 19 subunits. In the hippocampus, α5 subunits primarily coassemble with β3 and, in some cases, γ2, generating numerous subtypes with differential functional and pharmacological properties critical in learning and memory. The stoichiometry, structure, and gating of these subpopulations are poorly understood. Here we show using cryogenic electron microscopy and electrophysiology that the human α5β3 GABA_A_R predominantly assembles with 2α:3β stoichiometry, though a minority population of 1α:4β indicates multiple assemblies are possible. In a resting-like state, a conserved activation gate and Zn^2+^-coordination at histidines on β3 block ion conduction. Upon GABA binding, global rearrangements release Zn^2+^ and open the activation gate in nearly all receptors. The activated receptor is unaffected upon binding the anesthetic etomidate or anticonvulsant topiramate, supporting a conformational selection mechanism of action. This work thus reveals the assembly, activation, and modulation of a GABA_A_R subtype critical to cognition, providing templates for structure-based drug discovery.

## Abstract (web summary)

α5 subunit-containing GABAA are vital for learning and memory. Here, Cowgill et al. use cryo-EM and electrophysiology to study the α5β3 GABAA receptor subtype, giving insights into assembly, gating, and modulation by anesthetics and anticonvulsants.

## Introduction

Type-A γ-aminobutyric acid receptors (GABA_A_Rs) are pentameric ligand-gated ion channels (pLGICs) that drive the majority of inhibitory neurotransmission in the brain by passing a hyperpolarizing chloride current in response to the neurotransmitter GABA. These receptors control a diverse range of physiological processes through both phasic and tonic inhibition mediated by distinct populations of synaptic and extrasynaptic receptors, respectively^1,2^. GABA_A_Rs are critical drug targets, with distinct subtypes underlying key pharmacological effects. For instance, general anesthetics like etomidate are known to act through a range of GABA_A_R subtypes^3,4^; moreover, etomidate blocks memory formation even at subtherapeutic doses that do not induce loss of consciousness^5–7^. Subtype-specific inhibitors and genetic knockouts have attributed this amnesic effect of etomidate to GABA_A_Rs containing the α5 subunit, which is expressed particularly in the hippocampus both synaptically and extrasynaptically^8–10^. Similarly, the anticonvulsant topiramate has been shown to suppress cognitive function in addition to seizures^11^. Topiramate particularly potentiates at the low levels of GABA expected in extrasynaptic regions, where α5-containing subtypes have been shown to mediate tonic inhibition in hippocampus^12–14^. Based on clinical observations, such as these, α5 activity appears to underlie adverse cognitive effects of several GABAergic drugs; conversely, α5-specific modulators constitute promising drug candidates for conditions associated with learning and memory deficits like Down syndrome, Alzheimer’s disease, depression and schizophrenia^15^.

There are 19 genes encoding GABA_A_Rs subunits in humans (α1–6, β1–3, γ1–3, δ, ε, ρ1–3, π, and θ), each contributing to an N-terminal extracellular domain (ECD) with ten β-strands (β1–β10), and to a C-terminal transmembrane domain (TMD) with four membrane-spanning helices (M1–M4)^16^. As in other pLGICs, a poorly conserved sequence between the M3 and M4 helices contributes to an intracellular domain that may be disordered, at least in the absence of intracellular trafficking or signaling factors^17^. The full receptor consists of five subunits, with the five M2 helices lining a transmembrane ion-conduction pathway. GABA binding is thought to induce conformational changes that open an activation gate near the middle of the TMD, after which the channel desensitizes by contracting the intracellular-facing pore^18^. To initiate this process, GABA typically binds at a so-called orthosteric ECD site formed by loops A–C on the principal face of a β subunit, and by loops D–F on the principal face of a β subunitβ subunit, and by the complementary face of an α subunit; therefore, a minimal combination of α and β subunits is typically required to form functional GABA_A_Rs. Further incorporation of at least one γ subunit is associated with benzodiazepine sensitivity and clustering at synapses; αβγ combinations account for the majority of GABA_A_R structures reported thus far, though alternative assemblies are likely to be especially critical for extrasynaptic processes^19^. For the α5 subunit, native coexpression patterns and recombinant-cell electrophysiology indicate coassembly with β3 and, in some cases, γ2 subunits, generating multiple potential subtypes with differential functional and pharmacological properties^20–23^. For example, α5β3 receptors are susceptible to block by zinc ions (Zn^2+^), while α5β3γ2 receptors are sensitive to benzodiazepines^21^. GABA_A_Rs containing two or three subunit types are largely thought to assemble with 2:3 α:β or 2:2:1 α:β:γ stoichiometry, respectively^24^; however, corresponding structures have yet to be reported in the context of α5.

Driven by the therapeutic potential of α5-containing GABA_A_Rs, there is substantial interest in resolving structural determinants of their gating and pharmacology. Soon after the resolution revolution in cryogenic electron microscopy (cryo-EM), a single structure of a GABA-bound α5β3 receptor was reported in an unexpected 1:4 α:β stoichiometry, leaving open questions as to assembly preferences, gating transitions and drug binding in this subtype^25^. Chimeric studies have reported complex structures that combine elements of α5 with more biochemically tractable subunits, such as β3 or γ2, shedding light on potential binding modes of neurosteroids and benzodiazepines^26,27^. However, as these engineered constructs lack the capacity to bind GABA, they offer little insight into mechanisms of activation or modulation. A structure of the related α1β3 GABA_A_R was recently determined in the presence of GABA and/or various inhibitors, with the anticipated 2:3 α:β stoichiometry; however, the pore remained in a resting-like conformation even in the presence of agonist, revealing little about channel gating at other diheteromeric subtypes^25,28^. Thus, there are critical gaps in our structural understanding of α5β3 GABA_A_R subunit assembly and response to the absence or presence of agonist or modulatory drugs.

Here, we report eight cryo-EM structures of a human α5β3 GABA_A_R in the context of multiple membrane mimetics, stoichiometries and ligands. Combined with oocyte electrophysiology of wild-type and modified complexes, these structures address open questions as to α5β3 GABA_A_R assembly and gating, and reveal its mechanism of potentiation by the common therapeutics etomidate and topiramate. The predominant stoichiometry in our hands is 2:3 α:β, with a minority class of 1:4 α:β observable in our largest dataset. GABA binding restricts the mobility and extension of loop C, and extensively drives α5β3 into an activated-desensitized state, in evident contrast to α1β3 GABA_A_Rs. Etomidate and topiramate bind lipid-facing cavities formed particularly at β-α interfaces during channel activation, sharing an apparent conformational selection mechanism with implications for targeted drug design.

## Results

### Structures of human α5β3 GABA_A_Rs with predominant 2:3 α:β stoichiometry

**Fig. 1:**
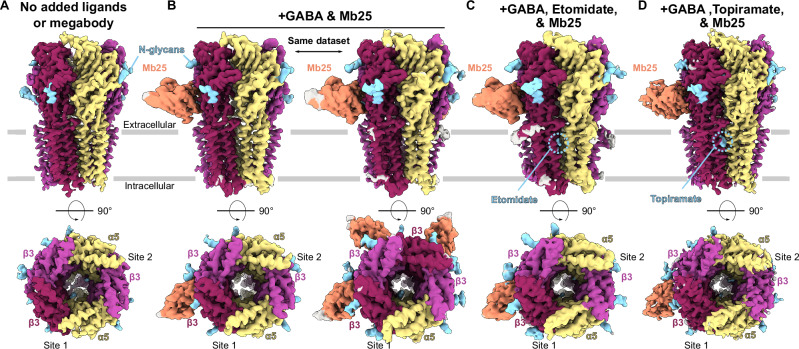
Structures of human α5β3 GABA_A_Rs with predominant 2:3 α:β stoichiometry. **A** Cryo-EM density map for α5β3-EM in micelles, resolved in a resting-like state without addition of any small-molecule ligand or megabody at freezing, viewed from the membrane plane (top) or from the extracellular side (bottom). Two α5 subunits (yellow) and three β3 subunits (magenta) are distinguishable by their patterns of N-linked glycosylation (blue). For clarity, the complementary β3 subunit at the β:β interface is shaded. Circled numbers in the extracellular view indicate orthosteric sites 1 and 2 as designated in this work. **B** Densities for α5β3-EM in nanodiscs, resolved in an apparent desensitized state upon addition of Mb25 and GABA, viewed and colored as in (**A**). As confirmed by the presence of Mb25 (salmon) at β3:β3 interfaces, two distinct classes obtained from this grid correspond to 2:3 (left) and 1:4 (right) α:β stoichiometries, representing 72% and 28% of resolved particles, respectively. **C** Density for α5β3-EM in nanodiscs, resolved in the desensitized state upon addition of Mb25, etomidate and GABA, viewed and colored as in (**B**). Densities attributed to etomidate (blue, dashed circle) occupy a TMD pocket at the β-α interface. **D** Density for α5β3-EM in nanodiscs, resolved in the desensitized state upon addition of Mb25, topiramate and GABA, viewed and colored as in (**B**). Densities attributed to topiramate (blue, dashed circle) occupy a TMD pocket at the β-α interface and, more weakly, the pore. Sample conditions are reported in “Methods” and Table 1.

To elucidate the stoichiometry of assembly, as well as mechanisms of gating and potentiation, of the human α5β3 GABA_A_R, we determined cryo-EM structures with various ligands in both lauryl maltose neopentyl glycol (LMNG) micelles and brain-lipid/saposin nanodiscs (Fig. 1, Supplementary Figs. 1 and 2, Tables 1, 2). In accordance with previous studies on αβ GABA_A_Rs^25,28^, and to improve biochemical tractability, we first removed the intracellular domains (M3–M4 loops) of the α5 and β3 subunits and inserted affinity tags at the N- and C-termini, respectively (Supplementary Fig. 3). To enable tracking of each subunit by fluorescence-detected size-exclusion chromatography (FSEC), we also added superfolder GFP (sfGFP) to the N-terminus of α5 and inserted mKalama into the M3–M4 loop of β3, producing a diheteromeric complex hereafter called α5β3-EM. Presumably due to the glycosylation of α subunits in the extracellular vestibule, we were able to use a single affinity purification step targeting the tag on our α5 construct without detecting any population containing more than two α5 subunits. The affinity-purified product from HEK cells coexpressing our α5 and β3 constructs eluted as a monodisperse peak by FSEC in either micelles or nanodiscs, and contained both subunits as indicated by the co-elution of sfGFP and mKalama signals (Supplementary Fig. 4). Density maps from α5β3-EM showed a clear distinction between α5 and β3 subunits based on differential glycosylation patterns. For an initial dataset collected from α5β3-EM in micelles, without adding either small molecules or antibody fragments at grid freezing, symmetry expansion followed by iterative focused classification on opposing ECDs enabled reconstruction in one predominant class (Fig. 1, Table 1, and Supplementary Fig. 1). The corresponding structure resolved to 3.14 Å with two α5 and three β3 subunits and occupied a resting-like state as described in detail below. A dominant 2:3 α5:β3 stoichiometry was further confirmed by the addition of the megabody Mb25, targeting the β3:β3 interface, in subsequent GABA-bound datasets^29^. A minority class of 1:4 α5:β3 was also observed in a large dataset (47,000 micrographs) collected in the presence of Mb25 and GABA, accounting for 28% of particles resolved on that grid. Additional GABA-bound structures in micelles or nanodiscs and upon extended incubation with GABA, etomidate or topiramate, were resolved to 3.10–3.81 Å and detailed below (Tables 1 and 2).

**Table 1:** Cryo-EM data collection, refinement, and validation statistics

Sample grid	Micelles	Micelles + Mb25, GABA	Nanodiscs + Mb25,GABA	
Resolved complex	2:3 + Zn^2+^ 9HAA	2:3 + Mb25, GABA 9HNQ	2:3 + Mb25, GABA 9HUM	1:4 + Mb25, GABA 9HNR
Data collection and processing				
Magnification	130,000	130,000	130,000	130,000
Voltage (kV)	300	300	300	300
Electron exposure (e^–^/Å^2^)	58.65	58.65	61.536	61.536
Defocus range (μm)	0.8–1.8	0.8–1.8	0.8–1.8	0.8–1.8
Pixel size (Å)	0.65	0.65	0.648	0.648
Symmetry imposed	C1	C1	C1	C1
Initial particle images	285,325	707,771	1,616,159	1,616,159
Final particle images	68,589	192,171	411,902	157,080
Map resolution (Å)	3.14	3.81	3.35	3.17
FSC threshold	0.143	0.143	0.143	0.143
Refinement				
Initial models used	2α, 3β AF2 models	2α, 3β AF2 models	2α, 3β AF2 models	1α, 4β AF2 models
Model resolution (Å)	3.26	3.86	3.43	3.26
FSC threshold	0.5	0.5	0.5	0.5
Map sharpening *B* factor (Å^2^)	−39.59	−80.53	−69.75	−84.71
Model composition				
Non-hydrogen atoms	14,256	15,211	15,174	17,170
Protein residues	1698	1821	1816	2056
Ligands	39	38	38	44
B factors (Å^2^)				
Protein	71.78	84.54	74.34	106.22
Ligand	80.95	83.35	88.35	107.53
R.m.s. deviations				
Bond lengths (Å)	0.003	0.013	0.003	0.003
Bond angles (°)	0.595	0.727	0.552	0.653
Validation				
MolProbity score	1.15	1.33	1.45	1.41
Clashscore	3.59	3.93	5.70	4.73
Poor rotamers (%)	0.07	0.06	0.00	0.00
Ramachandran plot				
Favored (%)	98.21	97.17	97.27	97.08
Allowed (%)	1.79	2.83	2.73	2.92
Disallowed (%)	0	0	0	0

**Table 2:** Cryo-EM data collection, refinement and validation statistics for samples with long application of GABA and/or drugs

Sample grid	Nanodiscs + Mb25, GABA (long)^a^	Nanodiscs + Mb25, etomidate, GABA	Nanodiscs + Mb25, topiramate, GABA (long)^a^	
Resolved complex	2:3 + Mb25, GABA 9HNS	2:3 + Mb25, GABA, etomidate 9HNT	2:3 + Mb25, GABA, topiramate 9RL5	2:3 + Mb25, GABA 9RPB
Data collection and processing				
Magnification	130,000	130,000	130,000	130,000
Voltage (kV)	300	300	300	300
Electron exposure (e^–^/Å^2^)	50	40.01	50	50
Defocus range (μm)	0.8–1.8	0.8–1.8	0.6–1.8	0.6–1.8
Pixel size (Å)	0.648	0.648	0.65	0.65
Symmetry imposed	C1	C1	C1	C1
Initial particle images	661,884	619,919	451,821	451,821
Final particle images	161,171	115,926	90,532	96,876
Map resolution (Å)	3.10	3.32	3.14	3.14
FSC threshold	0.143	0.143	0.143	0.143
Refinement				
Initial model used	2α, 3β AF2 models	2α, 3β AF2 models	2α, 3β AF2 models	2α, 3β AF2 models
Model resolution (Å)	3.22	3.46	3.26	3.30
FSC threshold	0.5	0.5	0.5	0.5
Map sharpening				
* B* factor (Å^2^)	−59.24	−61.37	−57.91	−56.89
Model composition				
Non-hydrogen atoms	15,174	15,197	15,218	15,127
Protein residues	1816	1815	1816	1811
Ligands	38	40	40	38
B factors (Å^2^)				
Protein	82.20	76.11	92.45	92.10
Ligand	81.49	101.10	106.27	93.90
R.m.s. deviations				
Bond lengths (Å)	0.003	0.014	0.012	0.012
Bond angles (°)	0.602	0.818	1.057	1.020
Validation				
MolProbity score	1.22	1.36	1.54	1.46
Clashscore	3.81	4.50	6.22	6.92
Poor rotamers (%)	0.00	0.00	0.00	0.00
Ramachandran plot				
Favored (%)	97.7	97.26	96.82	97.65
Allowed (%)	2.23	2.62	3.18	2.29
Disallowed (%)	0	0.11	0	0.06

^a^GABA (long) indicates incubation with GABA, as well as Mb25 and any other ligands, for 45 min prior to grid freezing. Where not specified, GABA was applied immediately (~15 s) prior to freezing.

To assess the functional gating and stoichiometry of wild-type and modified GABA_A_Rs (α5β3-WT and α5β3-EM, respectively), we measured GABA responses in *Xenopus laevis* oocytes coinjected with RNA encoding α5 and β3 subunits. Our α5β3-EM complex retained functional properties of α5β3-WT, including activation by micromolar GABA and inhibition by micromolar Zn^2+^, though the apparent affinity for GABA was reduced ~4-fold (EC_50_ 6.97 μM versus 1.84 μM) (Supplementary Fig. 4E, F). For α5β3-WT, GABA concentration responses could be curve-fitted with a Hill slope of 1.69 (95% confidence interval 1.38–2.11). While the Hill slope is not a direct measure of the number of ligand binding sites, it is generally considered a lower bound for the number of sites present; a value greater than 1 thus supports the presence of two GABA binding sites, consistent with a stoichiometry of 2:3 α:β, while the 1:4 α:β population would contain only one β-a interface capable of binding GABA^30^. Similar characterization of α5β3-EM yielded a Hill slope of 1.83 (95% confidence interval 1.49–2.28), indicating that cooperative gating was retained in our EM constructs. We focus the majority of our structural analyses below on the receptors with 2:3 stoichiometry.

### GABA drives α5β3 into the desensitized state

**Fig. 2:**
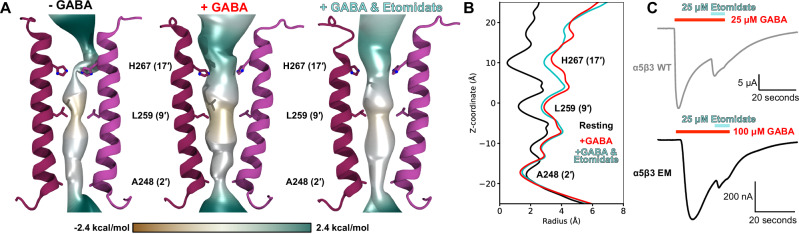
GABA drives α5β3 into the desensitized state. **A** Structural overlay of pore pathways (surface, colored by increasing hydrophilicity according to scale bar) with opposing β-subunit M2 helices (magenta) in α5β3-EM structures without GABA (left, PDB ID 9HAA), with 2 mM GABA (center, PDB ID 9HNQ) and with 200 μM etomidate + 500 μM GABA (right, PDB ID 9HNT). Key pore-facing residues at the presumed Zn^2+^ site (17′), activation gate (9′) and desensitizing constriction (2′) in M2 are represented as sticks and labeled. **B** Pore radius as a function of z-coordinate relative to the 9′ activation gate in the structures without GABA (black, PDB ID 9HAA), with GABA (red, PDB ID 9HNQ) and with etomidate+GABA (cyan, PDB ID 9HNT) shown in (**A**). **C** Example electrophysiology traces for α5β3-WT (top, gray) and α5β3-EM (bottom, black) in response to a saturating pulse of GABA (25 and 100 μM, respectively) followed by addition of 25 μM etomidate and subsequent washout. Insets indicate current versus time scales.

In our structure of α5β3-EM prepared without Mb25 or other ligands at grid freezing, the transmembrane ion-conduction pathway is narrower than a dehydrated chloride ion (1.8 Å radius) at the middle and extracellular end of the TMD (Figs. 1A and 2A, B). Specifically, the pore is constricted by amino-acid side chains at the 9′ and 17′ positions, where prime (′) numbers are relative to a conserved basic residue at the intracellular end of the M2 helices (Supplementary Fig. 3). At the 9′ position, conserved leucine residues from all five subunits form a so-called activation gate which occludes resting-state conduction in classical models of pLGIC gating^31^. Given the absence of GABA in this structure and constriction of its activation gate, we assigned it to a resting-like state. At the 17′ position, histidine residues on the three β3 subunits also project into the pore and coordinate a non-protein density that further blocks the ion-conduction pathway (Fig. 2A, B, and Supplementary Fig. 5A, B). Given the capacity of Zn^2+^ to inhibit α5β3 and bind histidine residues, we assigned this ion to the pore density. Although we did not deliberately add Zn^2+^ to our α5β3 samples, it is a required component of expression media for HEK293-derived suspension cells, and likely to co-purify with resting α5β3^32^. Indeed, the pore profile in this state resembled that of the α1β3 GABA_A_R in an inhibited, presumed resting-like state, which also contains Zn^2+^ at the level of 17'^28^ (Supplementary Fig. 5C).

In structures with GABA as well as the fiducial Mb25, the pore is substantially dilated relative to the resting-like state, particularly at and above the activation gate (Figs. 1B–D,2A, B, Table 1, and Supplementary Fig. 1). Dilation of the extracellular end of each M2 helix outward from the pore axis relieves leucine occlusion at 9′; it also disrupts histidine coordination at 17′, and density attributable to Zn^2+^ is no longer observed. In contrast to expansion in the outward-facing pore, the intracellular end is relatively constricted in the presence of GABA, narrowing below 2 Å at the 2′ position (proline or alanine in α5 or β3, respectively). Based on the presence of GABA, expansion of the activation gate (to 3 Å radius), relief of Zn^2+^ block and contraction at the intracellular pore, these structures appeared to represent a desensitized state of α5β3. We also determined cryo-EM structures with the positive allosteric modulator etomidate in addition to GABA, or adding GABA for 45 min rather than immediately (~15 s) prior to grid freezing (Table 2 and Supplementary Fig. 1). However, neither drug enhancement nor prolonged agonist exposure produced substantial changes in conformation, supporting the annotation of this α5β3 state as fully desensitized (Fig. 2A, B, and Supplementary Fig. 5D, E). Indeed, these pore profiles recapitulated the α1β2γ2 GABA_A_R bound to GABA and etomidate, which has also been reported as desensitized^33^ (Supplementary Fig. 5F, G).

Our consensus GABA-bound state differs from previous structures of αβ GABA_A_Rs. A GABA-α5β3 structure (1:4 α:β in LMNG micelles) was previously annotated as open, while a GABA-α1β3 structure (2:3 α:β in brain-lipid/MSP2N2 nanodiscs) was described as pre-open or primed^25,28^. In our hands, desensitization did not depend on subunit stoichiometry, given that our minority 1:4 α:β class included a similar 2′ constriction as in our 2:3 α:β complexes (Supplementary Fig. 5D, E, H, I). We determined GABA-bound structures of α5β3 in micelles as well as nanodiscs, but obtained superimposable pore profiles, indicating that α5β3 conformation is not heavily influenced by the choice of membrane mimetics (Table 1 and Supplementary Figs. 1A and 5D, E). We were unable to resolve any additional states in our cryo-EM datasets, whether prepared with short or long GABA incubation, micelles or nanodiscs, or with allosteric modulators.

To assess the physiological relevance of this consensus GABA-bound state, we measured electrophysiological responses of α5β3-expressing oocytes to 15 s of saturating GABA, followed by the addition of 25 μM etomidate. For both α5β3-WT and -EM, GABA stimulated a rapid peak current that decayed with sustained treatment; subsequent co-application of etomidate induced a modest additional peak, which further decayed prior to washout (Fig. 2C). The peak current displacement by etomidate can be thought to represent the subset of receptors not opened or desensitized by GABA alone, and thereby available for drug activation^28^. By this model, we could approximate the fraction of receptors driven to the open or desensitized states by GABA alone—described here as the activation propensity—as the ratio of the GABA peak current (I_GABA_) to the sum of the GABA and etomidate peak currents (I_GABA_+I_Etomidate_) (Supplementary Fig. 5L). According to this analysis, over 80% of the α5β3-EM population was activated by GABA alone (Supplementary Fig. 5M): in contrast to previous observations from the α1β3 subtype^28^, few α5β3 particles should be expected in a primed or otherwise non-activated state on grids with saturating GABA. Furthermore, the decay of current responses upon sustained treatment with either GABA or etomidate was characteristic of channel desensitization, and indicates a substantial fraction of α5β3-EM complexes should desensitize in the brief (~15-s) window between adding agonist and freezing cryo-EM grids. Thus, the activated-desensitized state observed in our α5β3+GABA structures corresponds to the expected majority of particles on grids with saturating GABA.

### A compact, homogeneous state of the neurotransmitter-binding pocket upon GABA activation

In addition to their resting versus desensitized pores, structures of α5β3-EM in the absence versus presence of GABA were notably distinct in the ECD, particularly at the orthosteric sites. The α5β3 complex contains two extracellular β-α interface sites expected to bind GABA, one closer to the β-β interface (orthosteric site 1), the other opposite to it (orthosteric site 2) (Fig. 1). Interestingly, non-protein densities were observed at both of the orthosteric sites in resting-like as well as desensitized states (Supplementary Fig. 6A–C). We hypothesized that the corresponding densities in the resting-like structure represented butyrate, which was added to 5 mM during protein expression, and differs from GABA only by the absence of a primary amine (Supplementary Fig. 6A). To test this hypothesis, we measured electrophysiological responses of α5β3-expressing oocytes to ~EC_20_ GABA before and after co-application of millimolar butyrate. We found that butyrate reversibly inhibits GABA activation of both α5β3-WT and -EM, consistent with this ligand selectively binding the resting-like state (Supplementary Fig. 6B). Comparable densities in GABA-bound structures were modeled as GABA (Supplementary Fig. 6C).

**Fig. 3:**
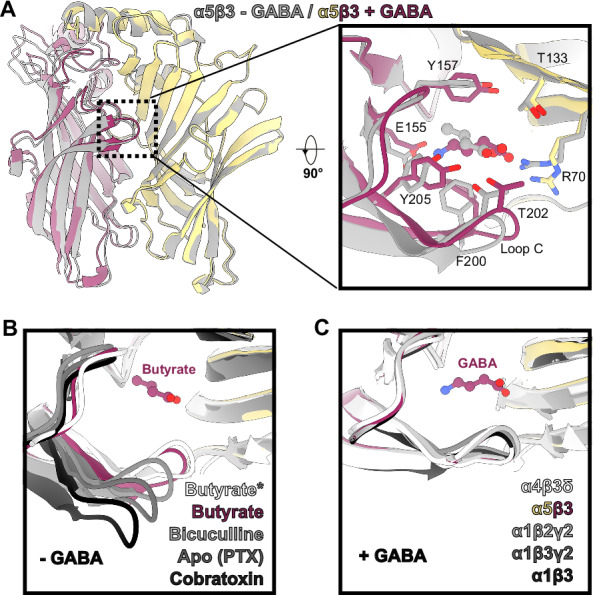
A compact, homogeneous state of the neurotransmitter-binding pocket upon GABA activation. **A** Neighboring β3- (magenta) and α5-subunit ECDs (yellow) surrounding orthosteric site 1 in GABA-bound α5β3-EM (PDB ID 9HNQ), viewed from the membrane plane. For comparison, resting-like α5β3-EM (gray, PDB ID 9HAA) is superimposed based on the complementary α5 subunit. Inset shows a zoom view of the orthosteric site, rotated 90° relative to the full-interface view (dotted box), with ligand-coordinating residues as sticks colored by heteroatom. GABA (magenta) and butyrate (gray) are shown as balls-and-sticks, also colored by heteroatom. **B** β3 (magenta) and α5 (yellow) at orthosteric site 1 in the resting-like structure of α5β3-EM, viewed as in (**A**) inset. For comparison, orthosteric sites in four other resting-like GABA_A_R structures are superimposed based on their complementary α subunits, including α4β3δ (white, PDB ID 7QN5), α1β2γ2 with bicuculline (light gray, PDB ID 6X3S), α1β3γ2 with PTX (dark gray, PDB ID 6HUG) and α1β3 with cobratoxin (black, PDB ID 7PC0). For clarity, the only atoms shown are for butyrate in α5β3-EM (magenta). Inset legend specifies bound ligands; for corresponding receptor subtypes, see inset legend in (**C**). *Although no ligand is modeled in the orthosteric site of resting-like α4β3δ, it is speculated to contain butyrate. **C** β3 (magenta) and α5 (yellow) at orthosteric site 1 in the GABA-bound structure of α5β3-EM, viewed as in (**B**). For comparison, orthosteric sites in four other GABA-bound structures are superimposed based on their complementary α subunits, including α4β3δ (white, PDB ID 7QN7), α1β2γ2 (light gray, PDB ID 6X3Z), α1β3γ2 (dark gray, PDB ID 6HUO) and α1β3 (black, PDB ID 7PBD). For clarity, the only atoms shown are for GABA in α5β3-EM (magenta).

Local changes in the orthosteric site of α5β3-EM could be visualized by superimposing resting-like and GABA-bound structures based on the complementary α5 subunit at a single β-α interface (Fig. 3A). A cluster of acidic and aromatic residues on the principal β3 subunit (E155 and Y157 on loop B, F200 and Y205 on loop C) rearranges to coordinate the GABA amino group, which is absent in butyrate. These interactions tighten the neurotransmitter-binding pocket, translocating the T202-Cα atom at the tip of loop C toward the bound ligand by 1.3 or 1.1 Å (in sites 1 or 2, respectively). T202, along with R69 and T132 from the complementary α5 subunit, also makes polar interactions with the carboxylate group of either butyrate or GABA. Aside from compacting the orthosteric site, loop C was better resolved in datasets with GABA, possibly reflecting stabilization in the presence of agonist (Fig. 1). To quantify this effect, we measured local scale in map regions around the orthosteric site using OccuPy^34^ (Supplementary Fig. 6D). Local scale is a normalized measure of contrast degradation in local regions of a density map, with lower values indicating heterogeneity due to structural flexibility, variable occupancy and/or particle misalignment. In the absence of GABA, map densities around the orthosteric sites and ECD periphery exhibited relatively low local scale values compared to other regions. GABA binding increased local scale throughout the ECD, particularly around loop C. Despite differences in overall resolution, local scale values were comparable for GABA-bound samples prepared in either micelles or nanodiscs. These measurements support a stabilizing as well as compacting effect of GABA on loop C, locking it down over the orthosteric site as a likely early step in receptor activation.

Relative flexibility of loop C in the absence of GABA was further substantiated by comparing α5β3-EM to previous GABA_A_R structures. Among five distinct heteromeric GABA_A_R assemblies in resting-like states, loop C assumed a range of poses, all relatively unlocked over the neurotransmitter-binding pocket (Fig. 3B). In this region, resting-like α5β3-EM was comparable to a recent α4β3δ-GABA_A_R structure, which similarly contained a serendipitous density attributable to butyrate^35^. The larger inhibitors bicuculline^33,36^ (at α1βXγ2-GABA_A_Rs) and cobratoxin^28^ (at α1β3) were associated with greater extension of loop C and expansion of the orthosteric site. Competitive inhibitors, such as these, are known to modulate α5-containing GABA_A_Rs^37,38^, despite poor accommodation in the compact site of our resting-like α5β3-EM structure, suggesting the neurotransmitter-binding pocket can actually sample a range of unlocked conformations. Indeed, one of the most expanded structures was with the pore blocker picrotoxin (PTX at α1β3γ2), where the orthosteric site appears to be unoccupied, indicating loop C can substantially extend when free from direct ligand interactions^36^. Conversely, poses of both loop C and GABA were superimposable among structures of the same heteromeric subtypes in the presence of agonist (Fig. 3C and Supplementary Fig. 6E). Activation is thereby associated with a relatively homogeneous as well as compact configuration of the orthosteric sites.

Structurally similar sites were possible at the α-β and β-β interfaces, but appeared to be less relevant than the orthosteric sites for α5β3 activation. The α-β interfaces exhibited negligible rearrangement upon GABA binding (Supplementary Fig. 7A). At the β-β interface, loop C adopted an extended pose in the resting-like state, similar to the β-α interface in PTX-inhibited α1β3γ2 GABA_A_Rs; in comparison, loop C was relatively locked down in the GABA-bound state (Supplementary Fig. 7B, C). Although this transition mimics those at orthosteric sites, it is likely attributable at least in part to Mb25 interactions with the β-β interface (Fig. 1B–D). A non-protein density was also evident at the β-β interface in both resting-like and GABA-bound α5β3-EM structures (Supplementary Fig. 6A, C). Consistent with its elongated profile and previous structures of β3δ-containing GABA_A_Rs^35^, we modeled this density as 2-hydroxyethylpiperazine-2-ethane sulfonic acid (HEPES) derived from protein purification buffers. The presence of HEPES in all electrophysiology and cryo-EM buffers made it an unlikely factor in gating comparisons in this work.

### Long-range conformational transitions implicated in GABA activation

**Fig. 4:**
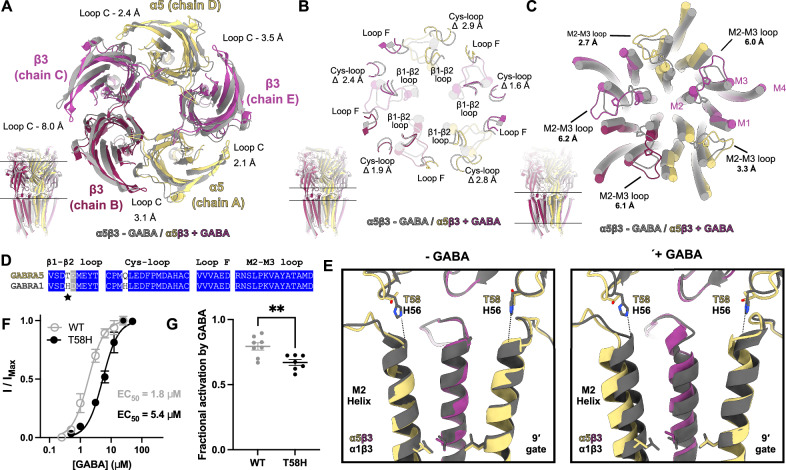
Long-range conformational transitions implicated in GABA activation. **A** Extracellular view of ECDs in GABA-bound α5β3-EM (PDB-9HNQ). Resting-like α5β3-EM (gray, PDB-9HAA) is superimposed based on TMD alignment. Distances indicate displacement of Cα-atoms at the tip of loop C (α5-T209 or β3-T202) between resting-like and GABA-bound states. Inset shows a side view of the receptor, highlighting the depicted slice. **B** ECD-TMD interface in GABA-bound and resting-like α5β3-EM. Changes (Δ) indicate increases in distance between Cα-atoms in the Cys-loop of subunit *i* (α5-P150 or β3-P144) and loop F of subunit *i* + 2 (α5-Q192 or β3-Q185), for the GABA-bound versus resting-like states. Main and inset figures are depicted as in (**A**). **C** TMD in GABA-bound and resting-like α5β3-EM. Distances indicate displacement of Cα-atoms at the midpoint of each M2–M3 loop (α5-P281 or β3-P273) between resting-like and GABA-bound states. Main and inset figures are depicted as in (**A**). **D** Alignment of α5- and α1-GABA_A_R sequences at the ECD-TMD interface. Conserved residues are highlighted in blue and star marks α5-T58/α1-H56. **E** Structures of α5β3-EM in the absence (left, PDB-9HAA) or presence (right, PDB-9HNQ) of GABA, highlighting β1–β2 loops and M2-helices of three subunits. View is from the membrane plane and colored as in (**A**). Structures of α1β3 GABA_A_Rs (gray) in the absence (left, PDB-7PC0)28 or presence (right, PDB-7PBD)28 of GABA are superimposed based on the highlighted regions. Residues on the β1–β2 loop and activation gate are shown as sticks and colored by heteroatom. Dashed lines indicate prospective hydrogen bonds between α1-H56 and the backbone of M2. **F** GABA concentration-response curves for α5β3-WT (gray) and ɑ5(T58H)β3 GABA_A_Rs (black). Data points represent mean normalized currents ± standard error of the mean for 7 independent oocytes. Wild-type data are reproduced from Supplementary Fig. 4E. **G** Fractional activation of α5β3-WT (gray) and ɑ5(T58H)β3 GABA_A_Rs (black) measured according to the protocol in Supplementary Figs. 5M and 8E. Solid bars represent mean ± standard error of the mean from 8 (α5β3-WT) or 7 (α5(T58H)β3) independent oocytes, also shown as individual measurements. Asterisks denote significance with *P* = 0.0046 in two-sided t-test.

In addition to locking down the orthosteric sites, GABA binding to α5β3-EM drove global conformational changes with implications for subtype-specific gating. GABA-induced transitions could be visualized by superimposing resting-like and GABA-bound structures based on the pentamer TMD (Fig. 4A–C). In this comparison, lockdown of loop C manifested grossly in rotation of the ECD counter-clockwise when viewed from the extracellular side: Cα atoms at the tip of each loop C displaced up to 8.0 Å toward their respective complementary subunits upon GABA binding, particularly in β-subunits (Fig. 4A). In parallel, the upper ECD contracted toward the z-axis, tightening connections between ECD subunits; this transition increased buried surface area (BSA) by 603.2 Å^2^ over all five ECD-ECD interfaces, especially those with a principal β-subunit (Fig. 4A and Table 3). Within each β subunit, gating transitions corresponded to a pivot of the ECD with respect to the TMD: as the upper ECD contracted, loops at the ECD-TMD interface moved outward, accompanying expansion of the TMD at and above the activation gate (Figs. 2A, B,4B, C, and Supplementary Fig. 8A). This expansion could be quantified based on conserved proline residues at the midpoint of each M2–M3 loop, whose Cα atoms displaced up to 6.2 Å away from the pore z-axis, again particularly in β3 subunits. Dilation of the TMD with respect to the z-axis loosened connections between TMD subunits, decreasing BSA by 932.9 Å^2^ over all five TMD-TMD interfaces, such that subunit-interface BSA decreased 318.9 Å^2^ overall (Fig. 4C and Table 3). TMD expansion was most evident at β-α subunit interfaces, where evolving cavities appeared to enable state-dependent ligand binding, as described in the next section.

**Table 3:** BSA (Å^2^) at subunit interfaces for resting-like and GABA-bound states in micelles

Interface (chain)	Resting-like (PDB ID 9HAA)			GABA-bound (PDB ID 9HNQ)			GABA-induced changes^a^		
	ECD	TMD	Full^b^	ECD	TMD	Full^b^	ECD	TMD	Full^b^
β(B)-α(A)^c^	1363.8	1288.5	2721.8	1573.5	1134.5	2780.4	209.7	−154	58.6
β(C)-β(B)^d^	1238.7	1372.6	2698.8	1426.9	1121.4	2651.1	188.2	−251.2	−47.7
α(D)-β(C)	1535.2	1190.6	2796.0	1538.6	1122.6	2720.1	3.4	−68	−75.9
β(E)-α(D)^e^	1424.8	1342.5	2837.9	1555.6	986.4	2616.4	130.8	−356.1	−221.5
α(A)-β(E)	1448.5	1188.1	2716.1	1519.6	1084.5	2683.7	71.1	−103.6	−32.4
Total	7011.0	6382.3	13770.6	7614.2	5449.4	13451.7	603.2	−932.9	−318.9

^a^GABA-induced changes are calculated by subtracting a given value for the resting-like state from that for the GABA-bound state. Positive and negative values indicate an increase and decrease, respectively, in BSA at a given interface upon GABA binding.

^b^BSA for the full subunit interface is marginally (<4%) greater than the sum of ECD and TMD interfaces, largely arising from protrusion of the principal M2–M3 loop towards complementary loop F (Fig. 4B).

^c^Orthosteric site 1 in the ECD, and modulate site 1 in the TMD, are located at the interface between chain-B β3 and chain-A α5.

^d^Mb25 binds at the ECD interface between chain-C and chain-B β3 subunits.

^e^Orthosteric site 2 in the ECD, and modulator site 2 in the TMD, are located at the interface between chain-E β3 and chain-D α5.

GABA induced more pronounced transitions in α5β3-EM compared to previous structures of the α1β3 GABA_A_R, potentially accounting for its differential propensity for activation^28^. As a proxy for extracellular rotation, the tip of loop C displaced up to 1.4 Å further in GABA-bound α5β3-EM versus α1β3; as a metric for transmembrane expansion, the midpoint of M2–M3 displaced up to 2.1 Å further (Supplementary Fig. 8B, C). These observations indicate that greater activation involves more extensive ECD twist and TMD dilation than is achieved in the GABA-bound α1β3 structure. Amino-acid sequences for α1 and α5 are largely conserved at ECD-TMD interfaces, including loop F and the β1-β2, M2–M3, and Cys-loops, offering limited insight into subtype-specific gating (Fig. 4D). One of the few deviations is a histidine (H56) in α1, which is substituted by threonine (T58) in α5. Located near the midpoint of the ECD β1–β2 loop, α1-H56 projects down toward the upper TMD, where it can donate an interdomain hydrogen bond to an unpaired backbone carbonyl at the C-terminus of M2. Such a contact is evident in structures of α1β3 and α1β2γ2 GABA_A_Rs, when determined with inhibitors in resting-like states^28,33^(Fig. 4E and Supplementary Fig. 8D). It is additionally retained in the GABA-bound structure of α1β3, determined in a primed state with a resting-like pore. Conversely, channel desensitization in the GABA-bound structure of α1β2γ2 dilates the upper TMD and disrupts the H56–M2 contact. In α5β3, T58 is too short to contact M2 in either resting-like or GABA-bound states, potentially reducing an energetic barrier to channel activation in this subtype. To test the functional relevance of this subtype-specific contact, we mutated α5-T58 to the α1-type histidine and coexpressed wild-type and mutant subunits with β3 in oocytes. GABA sensitivity decreased 3-fold in α5(T58H)β3 versus α5β3-WT receptors (EC_50_ 5.4 versus 1.8 μM, Fig. 4F). Following our previously described etomidate protocol (Fig. 2C), we also found the mutation reduced GABA activation propensity from 79 ± 2.8% to 67 ± 2.2% (Fig. 4G and Supplementary Figs. 5M and 8E). These results support a role for H56 in limiting GABA activation of α1-containing receptors, though additional, possibly distributed differences are also likely to contribute.

### Shared mechanism of potentiation by etomidate and topiramate

To elucidate mechanisms of allosteric modulation implicated in α5-dependent cognitive effects of common GABAergic drugs, we first inspected the structure of α5β3-EM bound to the general anesthetic etomidate, described above in the context of maximizing channel activation (Fig. 2). Whereas the structure was otherwise comparable to α5β3-EM with Mb25 and GABA alone, we observed non-protein densities at each β-α TMD interface, not evident in α5β3-EM structures without etomidate (Supplementary Fig. 9A, B). The equivalent site was unoccupied at α-β interfaces; a weaker density at the β-β interface in the etomidate structure suggested the presence of an additional binding site, perhaps with lower stability and/or occupancy, but could not be confidently built (Supplementary Fig. 9C). TMD resolution was generally more diffuse for the principal subunit at the β-β interface, indicating local heterogeneity that might compromise ligand resolution. As for the orthosteric sites, the α5β3 complex contains two transmembrane β-α interface sites capable of binding etomidate, one closer to the β-β interface (TMD site 1) and the other opposite to it (TMD site 2); chemical properties of these sites are detailed below. Protein as well as ligand densities were more clearly defined in TMD site 1 than 2, but both sites allowed for model building, producing ligands in comparable configurations that were reminiscent of etomidate structures with α1β2γ2^33^ α1β2γ2^33^ and homopentameric-β3 GABA_A_Rs^39^ as well as predictions from photoaffinity labeling^40^ (Supplementary Fig. 9D–F).

**Fig. 5:**
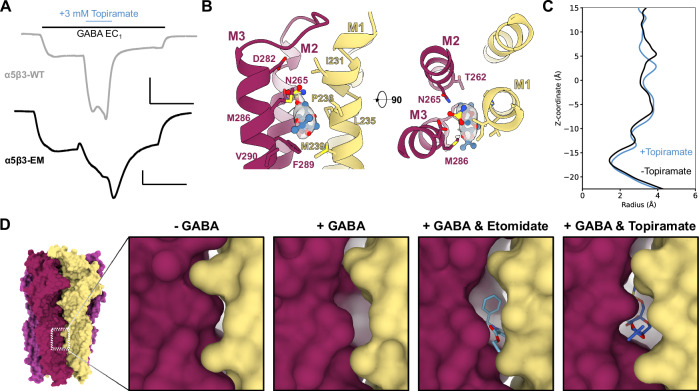
Shared mechanism of potentiation by etomidate and topiramate. **A** Example recording for α5β3-WT (gray) and α5β3-EM (black) in response to a short pulse of 3 mM topiramate applied during a 2-min application of GABA at EC_1_ (75 nM and 600 nM for α5β3-WT and -EM, respectively). Vertical scale bars represent 200 nA of current, and horizontal scale bars represent 30 s. **B** Topiramate binding in TMD site 2 of α5β3-EM (PDB ID 9RL5), showing M1–M2 of the α5 (yellow) and M2–M3 of the β3 subunit (magenta), viewed from the membrane plane (left) or the extracellular side (right). Topiramate (blue) is depicted as balls-and-sticks, with coordinating side chains as sticks, colored by heteroatom. Semitransparent surface indicates cryo-EM density corresponding to topiramate. **C** Pore profiles for α5β3-EM structures with (blue) and without evident topiramate binding (black), determined from the same dataset in the presence of topiramate as well as GABA and Mb25. **D** Surface representation of α5β3-EM, colored as in (**A**). Insets (left-to-right) show zoom views of TMD site 1 in structures determined in the resting-like state (PDB ID 9HAA), with Mb25 and GABA (PDB ID 9HNQ), with Mb25, GABA and etomidate (cyan, PDB ID 9HNT) or with Mb25, GABA and topiramate (blue, PDB ID 9RL5). Ligands are shown as sticks and colored by heteroatom.

Though similarly classified as a positive allosteric modulator of various GABA_A_Rs, the anticonvulsant topiramate is chemically distinct from etomidate and exhibits a complex pharmacological profile, including potentiation at low levels and block at high levels of GABA activation^12^. In our hands, EC_1_-GABA currents through both α5β3-WT and -EM were enhanced ~4-fold by 3 mM topiramate (Fig. 5A and Supplementary Fig. 10A). In a cryo-EM dataset for α5β3-EM with topiramate, one class of particles contained non-protein densities attributable to topiramate at β-α TMD sites 1 and 2 (Figs. 1D and 5B, Table 2, Supplementary Figs. 1A, 10B, C). Especially around the fructose diacetonide moiety of topiramate, these sites are largely hydrophobic, formed by side chains on the principal β3 M3-helix (M286, F289, and V290) and complementary α5 M1-helix (I231, L235, P236, and M239) (Fig. 5B and Supplementary Fig. 10D). The sulfonamide group is further coordinated by polar side chains on the β3 M2- (N265) and M3-helices (D282).

We found no evidence for topiramate binding at the α-β or β-β interfaces at similar thresholds. However, an additional diffuse density appeared in the outer mouth of the transmembrane pore, not apparent in structures with GABA alone or with etomidate (Supplementary Fig. 10F). Although the quality of this density was insufficient to confidently model topiramate in the pore, it localized around the 17′ position of the five M2-helices, similar to Zn^2+^ in the resting-like structure (Supplementary Figs. 5A–C and 10F). A secondary, lower-affinity binding mode for topiramate in the α5β3 pore was consistent with its electrophysiological profile, which included rebound spikes after drug washout, characteristic of open-pore block or stabilization of a desensitized state (Fig. 5A). Topiramate is relatively hydrophilic compared to etomidate (logP −0.8 versus 3.0), likely contributing to its capacity to bind in the water-filled pore, rather than exclusively in the membrane-facing TMD sites^41^.

Although our cryo-EM grids were prepared after incubating α5β3-EM in nanodiscs with 10 mM topiramate as well as GABA and Mb25 for 45 min, a second class in this dataset—representing 52% of resolved particles—contained no clear density attributable to the drug at the TMD sites, and less clear density in the channel pore (Table 2 and Supplementary Fig. 10C, F). Aside from loss of drug binding, this structure was of similar quality to the topiramate-bound structure (overall resolution 3.30 versus 3.26 Å), and pore profiles indicated they assumed comparable desensitized states (Fig. 5C and Table 2). No such class was identified in our etomidate dataset, despite collecting a larger number of initial particles. Transient occupancy of topiramate, even at millimolar concentrations, may account for some of its distinctive pharmacological features.

Structures in the presence of etomidate or topiramate assumed a desensitized state comparable to structures with GABA alone (Figs. 1B–D, 2A, B, and 5C). Whereas etomidate/topiramate sites at the β-α TMD interfaces were occluded in the resting-like state, expansion of the upper TMD upon GABA activation created accessible cavities; drugs bound in overlapping poses and induced little rearrangement on local or global scales (Figs. 4C, 5D, Table 3, and Supplementary Fig. 10C, E). These results contrasted with previous structures of α1β2γ2 GABA_A_Rs, which showed partial expansion of the upper TMD with GABA alone, and additional expansion upon addition of etomidate^33^(Supplementary Fig. 5J). For α5β3, both etomidate and topiramate appear to act by conformational selection, binding the β-α TMD sites in a state-dependent manner. This mechanism is at least partly subtype-specific, as homomeric-ρ1 GABA_A_Rs are largely insensitive to both etomidate and topiramate^12,42^ (Supplementary Fig. 10G). Mutating two residues on the principal face of ρ1 (I328, W349) to their equivalents in β3 (N265, M286) has been shown to confer etomidate sensitivity^42^. To test for a shared mechanism of topiramate potentiation, we measured electrophysiological responses of our previously reported ρ1-EM construct^43^ in the absence and presence of I328N/W349M mutations. In addition to increasing GABA sensitivity, mutations in ρ1-EM conferred enhancement by topiramate along with etomidate (Supplementary Fig. 10G–I). Indeed, superimposing the α5β3 complex onto GABA-bound ρ1-EM^43^ shows the relatively bulky ρ1-I328 and -W349 side chains would clash with topiramate, while mutating them to their β3 equivalents would create an accessible pocket (Supplementary Fig. 10J). Thus, structural and functional evidence support convergent conformational selection by etomidate and topiramate, subject to ablation via subtype-dependent sequence variations in the TMD sites.

## DISCUSSION

**Fig. 6:**
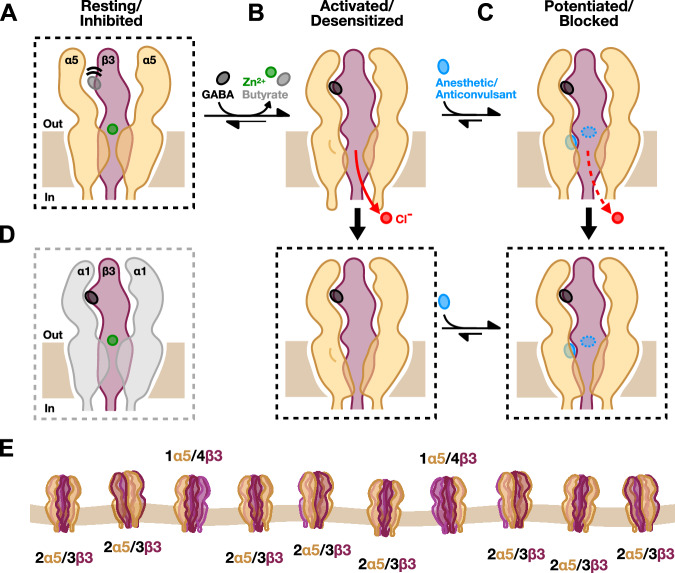
Mechanistic model of gating and allosteric modulation in ɑ5-containing GABA_A_Rs. **A** Schematic of a GABA_A_R containing ɑ5 (yellow) and β3 (magenta) subunits in a resting-like state, embedded in a plasma membrane (tan); the distal two subunits are hidden for clarity. This state features a relatively expanded, flexible ECD, and is capable of coordinating Zn^2+^ (green) in the outer transmembrane pore, as well as the inhibitor butyrate (gray) in the orthosteric ligand site at the extracellular β-ɑ interface. **B** Schematic as in (**A**) of structures induced by exposure to agonist, showing a presumably transient open state (top) capable of conducting Cl^–^ ions (red), and a longer-lived desensitized state (bottom) evident in cryo-EM preparations. GABA (black) displaces butyrate in the orthosteric site and stimulates conformational transitions that relatively stabilize, rotate and contract the ECD, dissociate Zn^2+^ and expand the transmembrane pore. **C** Schematic as in (**B**) of potentiated complexes, in which the general anesthetic etomidate or anticonvulsant topiramate (solid blue circle) selectively bind a site at the transmembrane β-ɑ interface in activated (open or desensitized) states. Partial occupancy of topiramate in the outer transmembrane pore (dashed blue circle) may account for inhibition at high levels of activation. **D** Schematic as in (**A**) of a GABA_A_R containing ɑ1 (gray) and β3 (red) subunits. GABA (black) induces only partial gating transitions and remains capable of coordinating Zn^2+^ (green) in the outer pore. **E** Alternative classes identified in a single ɑ5β3-EM grid indicate the potential for a mixed population of 2:3 and 1:4 ɑ:β assemblies. In **A**–**D**, black dashed boxes indicate structures reported in this work; the gray dashed box indicates a previously reported structure.

Our structural and functional characterization in this work supports a mechanistic model for the activation and potentiation of α5β3 GABA_A_Rs. In the resting state, the pore is constricted at an activation gate near the middle of the membrane plane; it is also prone to Zn^2+^ block via three histidine residues at the extracellular end of the three β subunits (Fig. 6A). Zn^2+^ inhibition is a distinguishing feature of αβ GABA_A_Rs, and plays a role in pathological conditions associated with increased excitability^44^; loss of Zn^2+^ sensitivity in αβγ GABA_A_Rs likely comes from the replacement of one β subunit with γ, which lacks a coordinating histidine. At rest, loop C is relatively extended and flexible over the orthosteric sites between β- and α-subunit ECDs; the receptor is accordingly susceptible to inhibitors covering a wide range of sizes, including butyrate and bicuculline. In contrast, GABA binding at the β-α ECD interface locks down loop C over the orthosteric sites (Fig. 6B). Associated rotation and compaction of the ECD is coupled to expansion of the TMD pore. The open state is sufficiently dilated to allow passive diffusion of anions, but transitions rapidly to a desensitized state in which conduction is blocked near the intracellular end; this state is captured in our GABA-bound cryo-EM structures, consistent with our electrophysiological recordings. Activation also expands cavities between M1 and M3 helices in neighboring subunits, enabling conformational selection by high-affinity etomidate or topiramate binding at β-α TMD interfaces (Fig. 6C). Higher concentrations of topiramate may bind a lower-affinity site in the channel pore. Activation of α5β3 contrasts with α1β3 GABA_A_Rs, which undergo lesser gating transitions and retain a Zn^2+^-blocked resting-like pore in the context of GABA binding, consistent with a lower propensity for agonist activation at this subtype^28^ (Fig. 6D).

Our cryo-EM structures and the cooperativity evident in our electrophysiology recordings support a predominant stoichiometry of 2:3 α5:β3 subunits. This result is consistent with previous electrophysiological studies in L929 and HEK cells, in which α5β3 concentration-response curves with Hill slopes above 1 indicated the presence of multiple GABA binding sites^21,45^. A preference for 2:3 α:β assembly also recapitulates recent structures of α1β3 GABA_A_Rs^28^. To minimize attrition during purification and other potential biases to the observed stoichiometry, we used a single affinity-purification step via tags on the α-subunits, enabled by the inability of α5 to form homopentamers. In a previous structure with predominant 1:4 α:β stoichiometry, α5β3 was purified via affinity tags on the β- and then α-subunits, potentially enriching for complexes with a higher proportion of β-subunits. In our hands, 1:4 α:β assemblies constituted a minor fraction of the α5β3 population (Fig. 6E). This work thus offers evidence for a single subtype assembling in multiple ratios, and supports the capacity of GABA_A_Rs to achieve functional diversity by forming a variety of complexes in vivo^35,46,47^. Still, given that our receptors were produced in a recombinant heterologous overexpression system, factors such as relative expression levels, association with scaffolding proteins and folding chaperones may perturb the native distribution of α5β3 assemblies. Further work is needed to clarify the stoichiometry and structure of related α5β3γ assemblies, which may be particularly relevant at hippocampal synapses^19^.

Our resting-like structure of α5β3-EM, obtained in the absence of fiducials, provides a framework for structural characterization of related receptors that may lack established antibodies or nanobodies suitable for cryo-EM. Thus, the structure grossly corresponded to anticipated features of the resting state, despite the appearance of a serendipitous density in the orthosteric site. This observation echoes a previous structure of the ɑ4β3δ subtype, where it was tentatively assigned to butyrate derived from cell expression media^35^. We demonstrate here that butyrate opposes activation of the receptor by GABA, supporting modeling of this ligand in our deposited structure. Butyrate lacks only the primary amine of GABA, so it is not surprising it can bind in the orthosteric site; however, it is roughly 4 times smaller than traditional inhibitors like bicuculline. The wide range in size of molecules that bind to this pocket indicates a high degree of flexibility in loop C at rest, consistent with the reduced resolution and decreased local scale from OccuPy analysis we observed at this site in the absence of GABA. Recent structures and molecular-dynamics simulations of a homomeric human ρ1-GABA_A_R similarly indicate that loop C is relatively flexible in the apo-resting state, but stabilized in the GABA-bound state, across the receptor family^43^. Future drug design efforts may leverage this dynamic orthosteric site using computational approaches like ensemble docking or AlphaFold 3 to improve specificity in competitive antagonists^48,49^.

Binding of HEPES at the β-β ECD interface is consistent with at least one previous structural study^35^. HEPES could be modeled in both the resting-like and GABAbound states of the receptor, despite substantial rearrangement upon binding of GABA and Mb25. The structure-function relevance, if any, of HEPES binding remains unclear, though the presence of this buffer in both our electrophysiology and cryo-EM buffers makes it an unlikely factor in transitions reported in this work. Given that histamine has also been demonstrated to bind this region and to activate both β3 homopentamers and α4β3δ GABA_A_Rs, the β-β interface may represent an intriguing new avenue for selectively targeting extrasynaptic GABA_A_Rs^35^.

In each of our GABA-bound α5β3 GABA_A_R structures, we obtained a single conformation corresponding to the desensitized state of the receptor. This observation is consistent with single-channel recordings of αβ GABA_A_Rs showing the open state in these receptors is short-lived, with open durations of less than 10 ms^21^. Unlike α1βXγ2 GABA_A_Rs, the global structure of α5β3 is preserved in micelles and nanodiscs, indicating this subtype is less sensitive to membrane composition^33,50^. Our functional recordings also showed GABA activates around 80% of α5β3 GABA_A_Rs without the requirement of positive allosteric modulators; this stands in contrast to α1β3 GABA_A_Rs, which have been shown to have a low propensity for activation and occupy a primed state even in the presence of GABA^28^.

Binding of etomidate or topiramate to α5β3-EM produced little structural change on a local or global scale, consistent with a fully activated-desensitized state of the GABA-bound structure and a shared conformational selection mechanism of drug potentiation. To our knowledge, our structures include the only currently available of any ligand-gated ion channel bound to the anticonvulsant topiramate, one of the 100 most common drugs prescribed in the USA^51^. Although its principal mechanisms of action remain unclear, topiramate has been shown to enhance receptor activity at low levels of GABA, but to block currents in the context of high GABA; this profile supports particular relevance to extrasynaptic receptor populations, such as those expected for α5β3, where they would primarily encounter low ambient GABA concentrations^12^. Open-state block by topiramate may reflect a general propensity of relatively hydrophilic modulators, given that the activated GABA_A_R pore is more polar than the membrane-facing TMD sites associated with potentiation. On the other hand, mechanisms of allosteric modulation may vary by receptor subtype: for example, synaptic α1β2γ2 GABA_A_Rs have been shown by substituted cysteine accessibility measurements to occupy a unique conformational state in the presence of general anesthetics^52^, and by cryo-EM to expand at the 9′ activation gate upon binding etomidate^33^. Given that the millimolar concentrations of topiramate used in our cryoEM studies are insufficient to saturate the binding sites in our structures, modulation of α5β3 receptors by topiramate is unlikely at therapeutic doses (<85 μM plasma concentration), but may be relevant in case of overdose or in carriers of mutations in α5 or β3 with altered properties^53^. Together with our observations of subunit assembly, subtype specificity, and modulatory mechanisms, this work thus offers a basis for biophysical insights and pharmacological targeting in the diverse and critical family of GABA_A_Rs.

## METHODS

### Sample preparation

To optimize biochemical tractability, our α5-EM construct incorporated the signal sequence from human α1 GABA_A_R, followed in sequence by sfGFP^54^, a Twin-Strep tag, and a recognition site for tobacco etch mosaic virus (TEV) protease^55^. Following the tags, the ECD+M1–M3 helices (residues 33–346) were separated from the M4 helix (residues 421–462) of human α5 (UniprotID: P31644) by a linker sequence (SQPARAA). Our β3-EM construct contained the coding sequence for human β3 (UniprotID: P28472) ECD+M1–M3 helices (residues 1–332), a linker containing the blue fluorescent protein mKalama1 (SQPGRA-mKalama1-GRAA), the β3 M4 helix (residues 447–473), followed by a two-residue linker (SR), a TEV protease site, and a 6-histidine tag. Aqueous GABA and etomidate stocks were prepared at 500 mM; topiramate stocks were prepared in dimethyl sulfoxide at 500 mM.

### Expression in oocytes and electrophysiology

*Xenopus* oocytes were purchased from Ecocyte Bioscience. To optimize expression in *Xenopus* oocytes, our α5-EM and β3-EM constructs, as well as our previously reported ρ1-EM construct^43^ were transferred into the pUNIV expression vector (Addgene Plasmid #24705)^56^. Mutations were introduced into α5 and ρ1-EM by methods analogous to QuikChange mutagenesis (Agilent). Plasmids encoding α5-WT, α5-T58H, α5-EM, β3-WT, β3-EM, ρ1-EM, and ρ1-EM/I328N/W349M GABA_A_R constructs were linearized using KpnI, and RNA was produced by in vitro transcription with the mMessage mMachine T7 Ultra transcription kit (Ambion) according to manufacturer protocols, including the tailing reaction. Stage-IV oocytes from *Xenopus laevis* frogs (Ecocyte Bioscience) were coinjected with 0.5 ng (WT background) or 10 ng (EM background) of RNA for each subunit. Injected oocytes were incubated for 1–2 days at 13 °C in post-injection solution (88 mM NaCl, 10 mM HEPES, 2.4 mM NaHCO_3_, 1 mM KCl, 0.91 mM CaCl_2_, 0.82 mM MgSO_4_, 0.33 mM Ca(NO_3_)_2_, 2 mM sodium pyruvate, 0.5 mM theophylline, 0.1 mM gentamicin, 17 mM streptomycin, 10,000 u/L penicillin, pH 7.5) before use in two-electrode voltage clamp measurements.

For recordings, glass electrodes were pulled and filled with 3 M KCl to give a resistance of 0.5–1.5 MΩ and used to clamp the membrane potential of injected oocytes at −70 mV with an OC-725C voltage clamp (Warner Instruments). Oocytes were maintained under continuous perfusion with Ringer’s solution (123 mM NaCl, 10 mM HEPES, 2 mM KCl, 2 mM MgSO_4_, 2 mM CaCl_2_, pH 7.5) at a flow rate of 1.5 mL/min. Buffer exchange was performed using a digital gravity-fed solution-exchange system (Scientific Instruments). Currents were digitized at a sampling rate of 2 kHz and lowpass filtered at 10 Hz with an Axon CNS 1440A Digidata system controlled by pCLAMP 10 (Molecular Devices).

To quantify GABA activation, concentration–response curves were fitted by nonlinear regression to the Hill equation below with variable slope using Prism 9.4 (GraphPad Software):

1 $$I/{I}_{{M}{a}{x}}=\frac{{[{\mathrm{GABA}}]}^{n}}{{[{\mathrm{GABA}}]}^{n}+{{{\mathrm{EC}}}_{50}}^{n}}$$

where *n* is the Hill slope and EC_50_ is the concentration of half-maximal activation. Each reported value represents the mean and the standard error of the mean for three to four oocytes.

To assess the extent of receptor activation with saturating GABA, we estimated the proportion in open and desensitized states as a fraction of the total receptor pool, defined here as the GABA activation propensity (Supplementary 5L). We adopted this metric as a variation on conventional measures of efficacy, i.e., the ability of a ligand to drive channels into the open state, given that we were unable to directly observe the open state in our cryo-EM datasets. To quantify activation propensity, we chose an electrophysiological protocol motivated by steady-state inactivation (h_∞_) measurements^57^. An initial pulse of saturating GABA was presumed to produce a variety of GABA-bound states, including resting-like/primed (pre-active) and open/desensitized (activated); further addition of etomidate, with continued saturating GABA, was presumed to drive all pre-active receptors into activated states. We also assumed that the number of receptors driven into activated states with a pulse of either GABA or GABA+etomidate was proportional to the change in current amplitude from the moment before the pulse to the peak during the pulse. Thus, to estimate the fraction of channels driven into activated states with GABA alone, we divided the current change elicited with GABA (I_GABA_) by the sum of the current changes induced with GABA and GABA+etomidate (I_GABA_+I_Etomidate_, Supplementary Fig. 5L). Since this analysis lumps together the open and desensitized states, it should not be heavily influenced by differences in the extent of desensitization between channel variants. It should also minimize confounds from kinetic effects of etomidate on rates of opening or desensitization^58,59^, which can overestimate the change in peak current between isolated GABA and GABA+etomidate pulses. Notably, this protocol approximates the fraction of channels still available for activation seconds after the application of GABA, which roughly corresponds to our timepoint of cryo-EM grid freezing.

### Baculovirus production

Baculovirus production was modified from previously described protocols^60^. Briefly, chemically competent DH10BacVSV cells (Geneva Biotech) were transformed with a modified pEZT-BM vector (Addgene Plasmid #74099) encoding either the α5-EM or β3-EM GABA_A_R subunit and incubated at 37 °C on transposition plates until the distinction between blue and white colonies was apparent^60^. White colonies for each subunit were picked and grown in suspension overnight, and bacmid DNA was isolated as described previously^61^. Bacmids encoding α5-EM or β3-EM GABA_A_Rs were used to transfect TriEX Sf9 cells (Novagen, cat #71023) in 6-well plates using Cellfectin II (Fisher) according to manufacturer instructions, except that 3 to 5 μg of bacmid was used. Supernatant containing the P1 virus was harvested following 96 h incubation at 27 C. The virus was amplified by infecting 30-mL suspension Sf9 cell cultures^61^. Due to the slow amplification of the virus in this step, it was necessary to continue splitting cells to maintain cell density between 1 and 4 × 10^6^ cells/mL until ~1% of cells showed red fluorescence due to the constitutive mCherry in the bacmid. Following this, cells were allowed to grow until the cultures were visibly red. P2 virus was further amplified by infecting 100 mL cultures of Sf9 cells to generate P3 virus that was used for transducing HEK cells. The structures obtained in this study were obtained from a total of three rounds of P3 virus production from the same P2 stocks.

### Expression and purification

Expi293F GnTl^–^ cells (Gibco, cat #A39240) were cultured in Expi293 Expression Medium (Gibco), and infected at a density of 3 × 10^6^ cells/mL with a 1.2:1 mixture of baculoviruses encoding the α5-EM and β3-EM subunits, respectively, at a 2.75% v/v virus/culture ratio. After 6 h incubation at 37 °C, 5 mM sodium butyrate was added to cultures, and flasks were moved to 30 °C. Cells were harvested 48 h post-transduction and washed once with phosphate-buffered saline before flash-freezing cell pellets in liquid nitrogen.

All steps of protein purification and grid preparation were performed at 4 °C or on ice. Cell pellets from 2 L culture were resuspended in 100 mL cell resuspension buffer (300 mM NaCl, 40 mM HEPES, pH 7.5 and 2 Complete protease inhibitor tablets (Roche)) and sonicated to lyse cells. Cell lysate was spun at 50,000 × *g* for 45 min to pellet crude membranes. The membrane pellet was resuspended in 100 mL 2X solubilization buffer (600 mM NaCl, 80 mM HEPES, pH 7.5 with 2 tablets of cOmplete protease inhibitor) and homogenized via sonication. Solubilization was initiated by the addition of 100 mL 2× detergent mixture (2% LMNG, 0.2% cholesteryl hemisuccinate (CHS)), and the mixture was stirred for 2 h and 30 min. Solubilized membranes were clarified by centrifugation at 50,000 × *g* for 45 min. The supernatant was applied to 4 mL Streptactin Superflow resin (IBA) that was previously equilibrated in buffer A (300 mM NaCl, 20 mM HEPES, 0.005% LMNG, 0.0005% CHS, pH 7.5), and samples were batch-bound for 90 min with gentle mixing. The resin was washed with 20 column volumes of buffer A and eluted using 5 column volumes of buffer A with 10 mM desthiobiotin (Sigma). The affinity-purified protein was concentrated and further purified by SEC on a Superose 6 increase 10/300 column (Cytiva) with a mobile phase of 100 mM NaCl, 20 mM HEPES, 0.005% LMNG, 0.0005% CHS, pH 7.5. Peak fractions were pooled and concentrated to ~3 mg/ml for grid preparation or nanodisc reconstitution.

### Nanodisc reconstitution

The SapA (saposin) expression plasmid was a gift from Salipro Biotech AB. Saposin was purified according to previously published protocols^62^. For the reconstitution of α5β3-EM into nanodiscs, purified α5β3-EM GABA_A_Rs, saposin and porcine polar brain lipids (Avanti) were mixed at the molar ratio 1:15:150. The mixture was incubated on ice for 1 h before Bio-Beads SM-2 resin (Bio-Rad) was added, and the mixture was gently shaken overnight at 4 °C. After ~16 h incubation with biobeads, the supernatant containing α5β3-EM/saposin/lipid nanodiscs was collected and further purified by SEC on a Superose 6 column with buffer containing 20 mM HEPES, pH 7.5, 100 mM NaCl. Peak fractions were pooled and concentrated to ~1 mg/mL.

### Megabody expression and purification

Mb25 was purified as described previously^29^. Cultures (500 mL) were grown to an OD of 2 in TB and induced overnight with 1 mM IPTG at 25 °C. Cells were harvested and resuspended in 80 mL suspension buffer (20% sucrose, 0.5 mg/mL lysozyme, 50 mM Tris, 1 mM EDTA, 150 mM NaCl, pH 8) and incubated at 4 °C for 30 min. Cell debris was pelleted at 8000 × *g* for 25 min. The supernatant containing periplasmic proteins was loaded onto 2 mL bed volume of Ni-NTA resin pre-equilibrated in wash buffer (10 mM Tris, 140 mM NaCl, 5 mM imidazole, pH 7.3). The column was washed with 15 mL wash buffer before elution in wash buffer supplemented with imidazole to a final concentration of 500 mM. Affinity-purified megabody was desalted by running a Superdex 200 SEC column in 140 mM NaCl, 10 mM Tris, pH 7.3. Purified protein was concentrated to a concentration of 4 mg/mL and flash frozen until use in grid preparation.

### Grid preparation and EM data acquisition

For the receptor without added ligands or Mb25 (resting-like), fluorinated foscholine 8 (FFC-8) (Anatrace) was added to 2 mM with LMNG-solubilized α5β3-EM GABA_A_R immediately prior to grid freezing. Subsequent samples prepared with Mb25, in micelles or nanodiscs, showed no preferred orientation even in the absence of FFC-8, and could be resolved at lower protein concentration (~1 versus ~3 mg/mL). Mb25 was added to samples at a final concentration of 0.4 mg/mL, corresponding to a 3-fold molar excess, and incubated 45 min until freezing. Structures with GABA as the only small-molecule ligand were prepared by adding GABA to 2 mM either ~15 s before freezing (LMNG and short-incubation datasets) or 45 min before freezing, together with Mb25 (long). For the structure with etomidate, nanodisc-reconstituted sample was incubated with 200 μM etomidate for 45 min along with Mb25, then mixed with 500 μM GABA ~15 s before freezing. For structures with topiramate, the nanodisc-reconstituted sample was incubated with 10 mM topiramate and 2 mM GABA for 45 min along with Mb25 before freezing. To prepare grids, pre-mixed samples (3 μL) were applied to glow-discharged R1.2/1.3 300 mesh Cu (topiramate) or 400 mesh Au (all others) grids (Quantifoil), blotted for 2 s with force 2 (topiramate) or 0 (all others) and plunged into liquid ethane using a Vitrobot Mark IV (Thermo Fisher Scientific) at 22 °C. Electron micrographs were collected at 300 kV using a Titan Krios (Thermo Fisher Scientific) electron microscope with a K3 Summit detector (Gatan) operating at ×130,000 magnification, corresponding to 0.648 or 0.65 Å/px, using the EPU automated collection software (Thermo Fisher Scientific). Micrographs were recorded over 1.1 to 1.4 s and fractionated into 40 frames for a total flux of ~40 to 65 e^–^/Å^2^ at the sample level as indicated in Table 1.

### Image processing

Dose-fractionated images in super-resolution mode were internally gain-normalized and binned by 2 in EPU during data collection. Beam-induced motion was corrected in Relion 4.0^63^. Contrast transfer functions were estimated for motion-corrected micrographs using CTFFIND4.1^64^, and particles were automatically picked using the internal model in Topaz 0.2.5^65^. Picked particles were binned 4 × 4 and subjected to two rounds of 2D classification in Relion 4.0 to remove empty nanodiscs and particles on carbon. Classes with recognizable channel features were re-extracted and centered by applying alignment offsets. These particles were used to generate initial models in CryoSPARC 4.0^66^. Further processing was done in Relion 5.0, including 3D classification without alignment to check the compositional and structural heterogeneity of classes. Multiple rounds of CtfRefine and polishing were used to improve resolution before a final refinement using Blush regularization^67^.

For the resting-like dataset, symmetry expansion was performed before final refinement to improve alignment of the heteromeric complex (Supplementary Fig. 1). Briefly, the particle stack was refined in C5 symmetry, then expanded 5-fold such that each particle was present in each of 5 possible alignments. Focused classification was then used to pull out particle replicates with ɑ5 subunits the Chain-A position, based on differences in glycosylation relative to β3 subunits. This subset of particle replicates was subjected to a second round of focused classification with a mask around the ECDs of Chains C and D. The class with a second ɑ5 subunit as Chain D gave the cleanest separation of differentially glycosylated subunits, and was used for final refinement following removal of a small fraction of duplicated particles (<10% of total remaining).

### Model building

Initial models of α5-EM and β3-EM monomers were generated using AlphaFold2 (AF2) by entering each engineered protein sequence into ColabFold v1.2.0^68^. Individual subunit models were fitted into cryo-EM densities and manually edited using Coot^69^. Crystallographic information files (cifs) for ligands were prepared from isomeric SMILES strings using Grade2^70^. Glycans were modeled based on previously assigned glycosylation patterns of α5 and β3^25^, removing unresolvable moieties on the vestibular α5 glycan. Final models were optimized using real-space refinement in PHENIX^71^ and validated by MolProbity^72^. Subunit interfaces were analyzed using the PDBePISA server^73^, and pore-radius profiles were calculated using the channel annotation package (CHAP) version 0.9.1 in Gromacs 2018^74^. Hydrophobic estimates were calculated using MLPP^75^. Structure figures were prepared using UCSF ChimeraX^76^ and VMD. Mechanistic models (Fig. 6) were created by the authors using Inkscape (Inkscape Project).

### Reporting summary

Further information on research design is available in the Nature Portfolio Reporting Summary linked to this article.

## Supplementary information

Supplementary Information

Reporting Summary

Transparent Peer Review File

## Source data

Source Data

## Data Availability

All unique/stable reagents generated in this study are available from the corresponding author, Erik Lindahl (erik.lindahl@scilifelab.se), without restriction. Cryo-EM density maps have been deposited in the Electron Microscopy Data Bank under accession numbers EMD-51980 (resting-like in micelles), EMD-52312 (GABA-bound in micelles), EMD-52416 (GABA-bound in nanodiscs), EMD-52313 (GABA-bound in nanodiscs, 1:4 α:β), EMD-52314 (GABA-bound in nanodiscs, long incubation with GABA), EMD-52315 (etomidate- and GABA-bound in nanodiscs), EMD-54031 (topiramate- and GABA-bound in nanodiscs, long incubation with topiramate and GABA), EMD-54151 (GABA-bound in nanodiscs, long incubation with topiramate and GABA). Model coordinates have been deposited in the Protein Data Bank under accession numbers PDB 9HAA (resting-like in micelles), 9HNQ (GABA-bound in micelles), 9HUM (GABA-bound in nanodiscs), 9HNR (GABA-bound in nanodiscs, 1:4 α:β), 9HNS (GABA-bound in nanodiscs, long incubation with GABA), 9HNT (etomidate- and GABA-bound in nanodiscs), 9RL5 (topiramate- and GABA-bound in nanodiscs, long incubation with topiramate and GABA), 9RPB (GABA-bound in nanodiscs, long incubation with topiramate and GABA) . Source data are provided with this paper.
