# Supplementary material for: Structural basis for activation and potentiation in a human α5β3 GABAA receptor
Source: Nat Commun. 2026 Jun 15;17:5289. doi: 10.1038/s41467-026-74279-3 (PMC13269941; doi:10.1038/s41467-026-74279-3)
Supplement: Supplementary file 1 — Supplementary Information [file 41467_2026_74279_MOESM1_ESM.pdf]

Supplementary

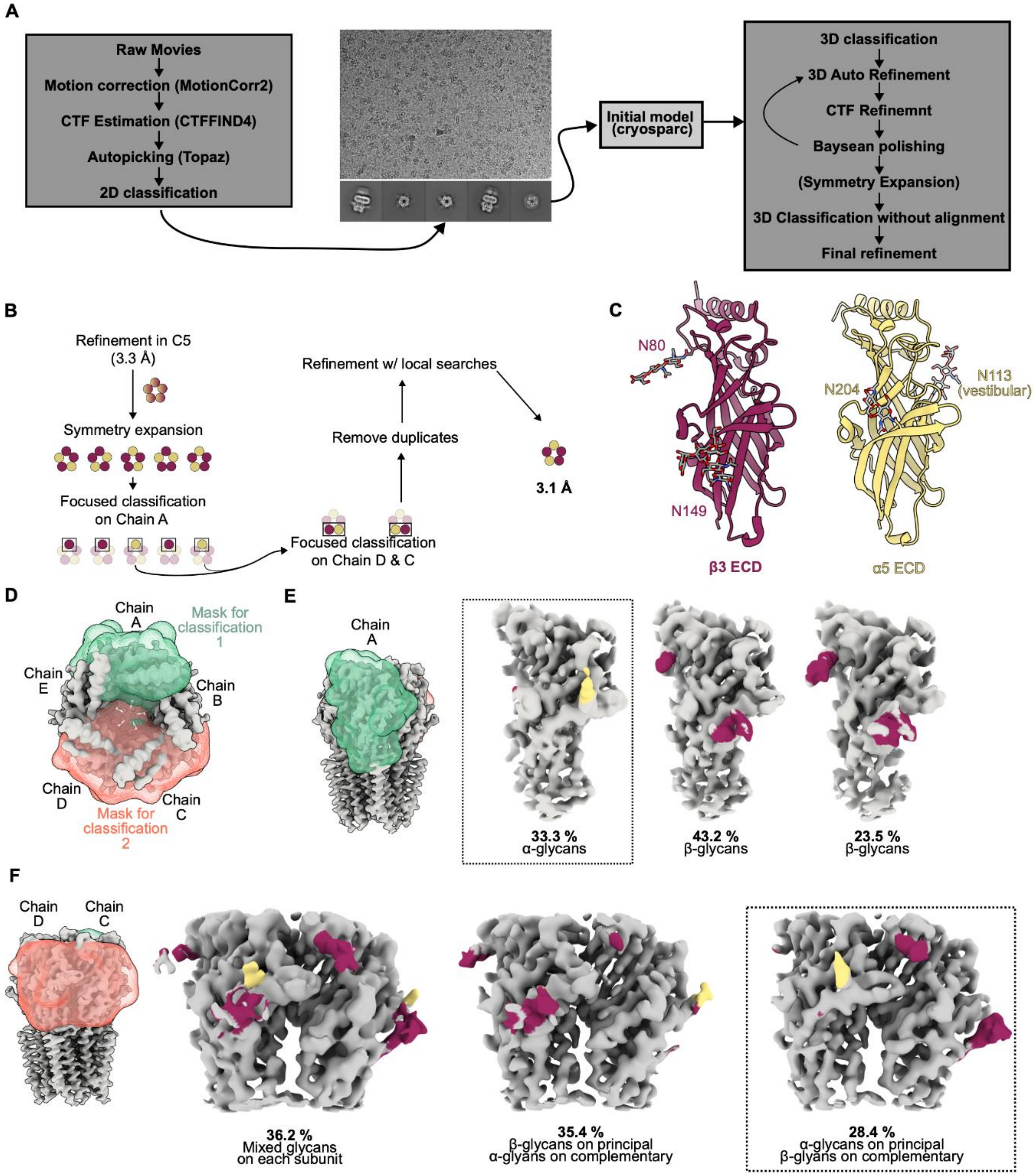

Supplementary Figure 1. Cryo-EM data-processing workflow for α5β3-EM structures.

(A) Generalized data-processing workflow for datasets in this study.

(B) Symmetry-expansion workflow for reconstruction of heteromeric receptors without fiducials, applied here to the structure in the resting-like state. The particle stack was first refined in C5 symmetry, then expanded 5-fold such that each particle was present in each of the 5 possible alignments, as shown by cartoons. Focused classification was then used to pull out particle replicates with α5 subunits the Chain A position, based on the differences in glycosylation relative to β3 subunits. This subset of particle replicates was subjected to a second round of focused classification with a mask around the ECDs of Chains C and D. The class with a second α5 subunit as Chain D gave the cleanest separation of differentially glycosylated subunits, and was used for final refinement following removal of a small fraction of duplicated particles (<10% of total remaining).

(C) Individual β3 (left, magenta) and α5 ECDs (right, yellow) showing the positions of glycosylation used to identify subunits during the focused-classification steps highlighted in panels d-f. Whereas most glycans faced the receptor surface, α5-N113 occupied the extracellular vestibule of the channel.

(D) Masks used for first (green) and second (salmon) iterative classification steps to align symmetry-expanded particles, overlaid with the initial C5 refinement (gray).

(E) First round of focused classification showing the mask used (left, green) and resulting classes (gray). Glycan densities are colored according to their linkage to either the α5 (yellow) or β3 subunit (magenta). Models shown in panel c were merged and fitted into the density maps. The first class (33.3% of particles) was selected, as it showed clear glycan density at the N204 position expected for α5, with little density near positions expected for β3.

(F) Second round of focused classification, with maps colored as in panel e. The third class, with α5 as Chain D and β3 as Chain C, was used for the final refinement. 'Mixed glycans' indicates density at both α5-specific (N204) and β3-specific (N80/N149) positions.

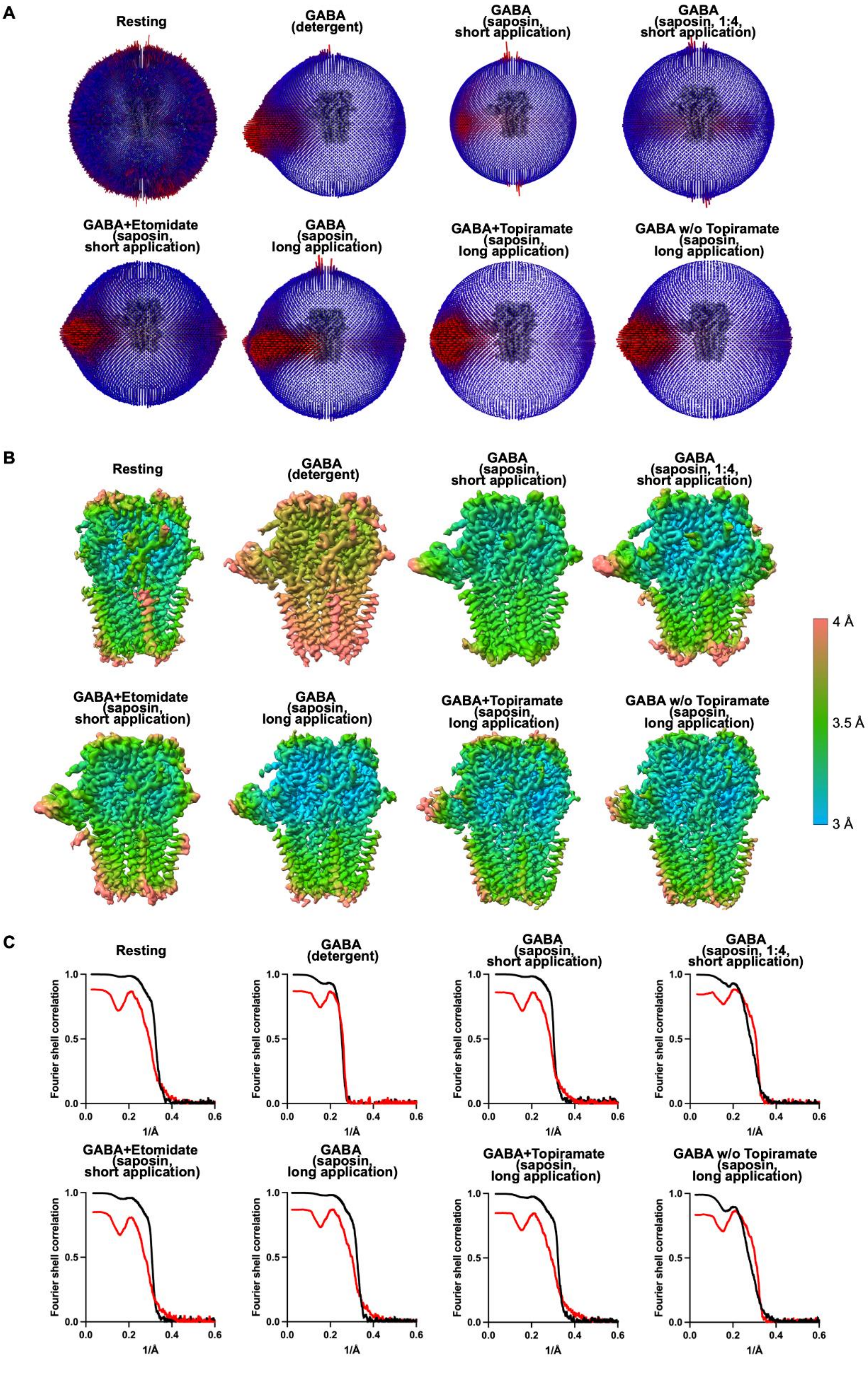
A
Resting
GABA (detergent)
GABA (saposin, short application)
GABA (saposin, 1:4, short application)
GABA+Etomidate (saposin, short application)
GABA (saposin, long application)
GABA+Topiramate (saposin, long application)
GABA w/o Topiramate (saposin, long application)
B
4 Å
3.5 Å
3 Å
C
Fourier shell correlation
1/Å
0.0
0.2
0.4
0.6
0.5
1.0

Supplementary Figure 2. Cryo-EM refinement parameters.

(A) Angular distribution maps for the final reconstructions used in this study.

(B) Local resolution of electron-density maps for each reconstruction.

(C) Map-to-map (black) and model-to-map Fourier shell correlation curves (red) for each reconstruction.

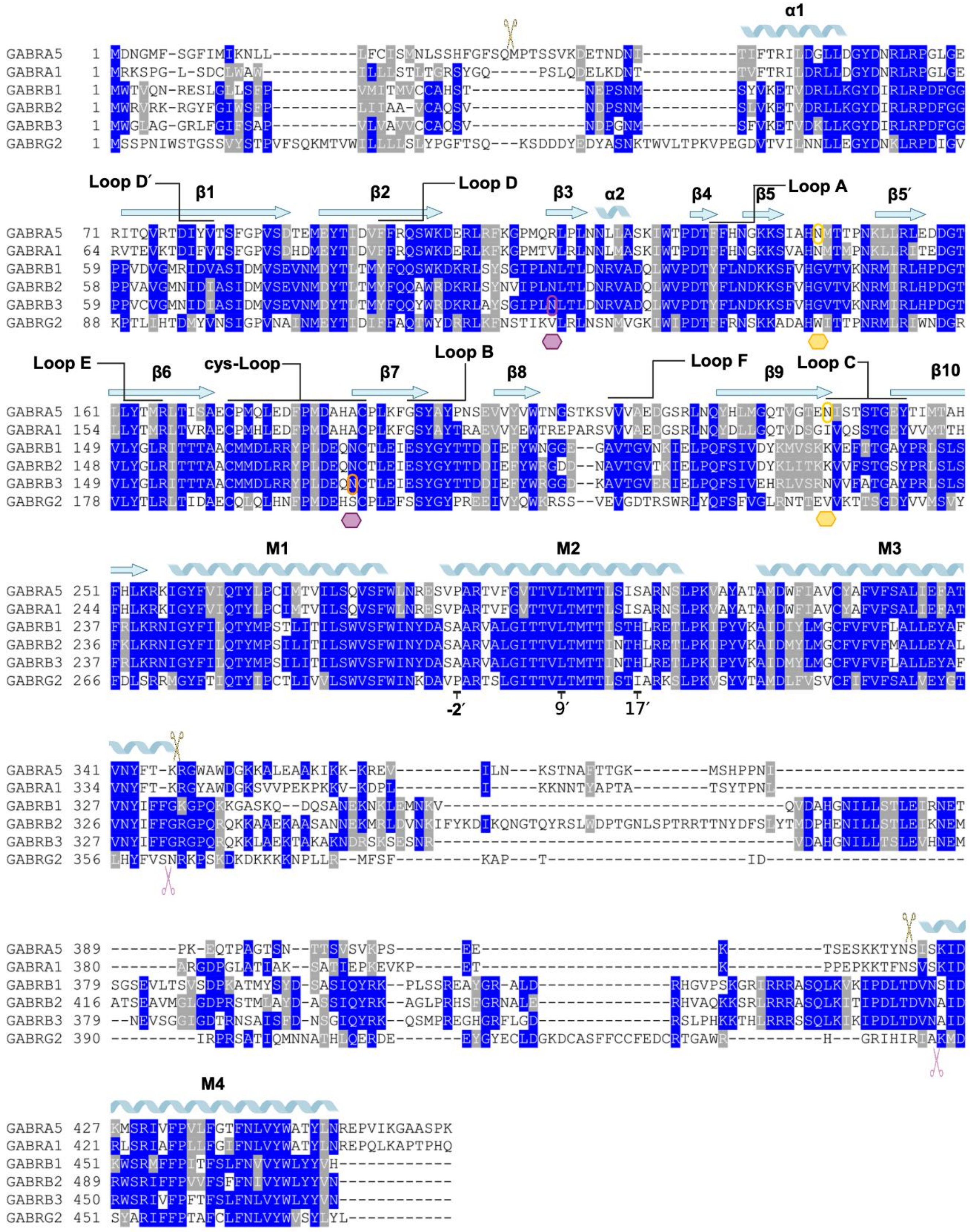

Supplementary Figure 3. Sequence alignment of relevant GABA$_A$R subunits.

Sequence alignment between α5, β3 and common coassembling GABA$_A$R subunits. Glycans specific to α5 (yellow) and β3 (magenta) are marked with hexagons. Scissors above the alignment indicate modification to the α5 sequence, while scissors below the alignment indicate modification to β3.

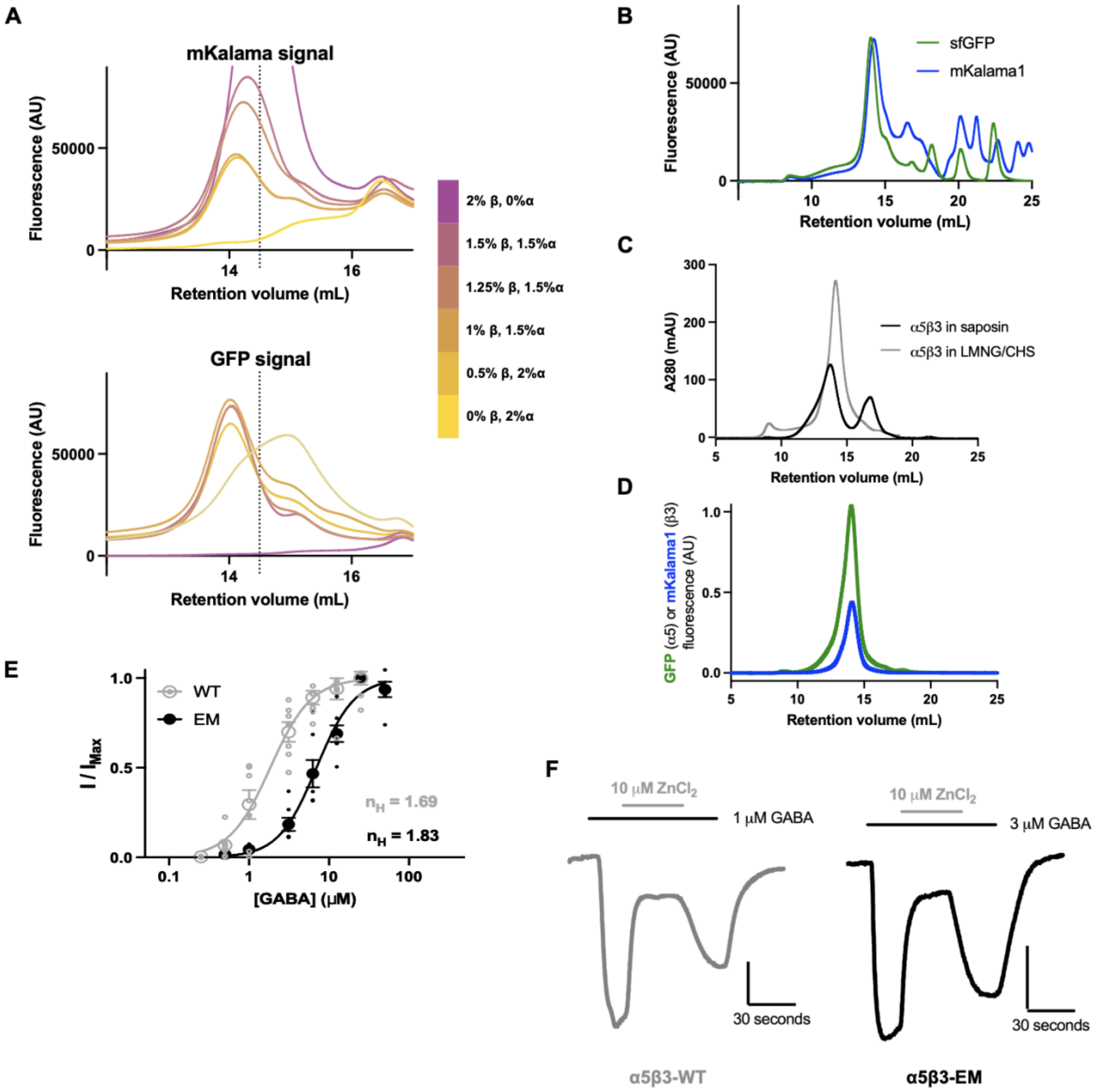

A
mKalama signal
Fluorescence (AU)
50000
0
14
16
Retention volume (mL)
GFP signal
2% β, 0%α
1.5% β, 1.5%α
1.25% β, 1.5%α
1% β, 1.5%α
0.5% β, 2%α
0% β, 2%α
B
sfGFP
mKalama1
10
15
20
25
C
A280 (mAU)
300
200
100
α5β3 in saposin
α5β3 in LMNG/CHS
5
D
GFP (α5) or mKalama1 (β3) fluorescence (AU)
1.0
0.5
0.0
E
I / I_Max
WT
EM
n_H = 1.69
n_H = 1.83
0.1
1
10
100
[GABA] (μM)
F
10 μM ZnCl2
1 μM GABA
3 μM GABA
30 seconds
α5β3-WT
α5β3-EM

Supplementary Figure 4. Biochemical and functional characterization of α5β3 $GABA_A$Rs.

(A) Titration of viruses used for transduction. Suspension cultures were transduced with six different ratios of α5-EM and β3-EM viruses, colored according to legend at right. Expression and assembly was then assessed by FSEC using detectors for mKalama-labeled β3-EM (top, 385 nm excitation, 456 nm emission) and sfGFP-labeled α5-EM (bottom, 480 nm excitation, 510 nm emission) to determine an optimal ratio of viruses to use in large-scale cultures. Fluorescence intensity is shown in arbitrary units (AU).

(B) FSEC profile of the condition used for large-scale expression of α5β3-EM, with the blue curve representing the fluorescence signal in the mKalama detector, and green the sfGFP detector. Fluorescence intensity is shown in arbitrary units (AU).

(C) SEC profiles of α5β3-EM purified in detergent (gray) or nanodiscs (black), monitored by UV absorbance at 280 nm. Absorbance values are reported in milli-absorbance units (mAU).

(D) FSEC profile of α5β3-EM in nanodiscs showing co-elution of sfGFP (green) and mKalama1 (blue), indicating a heteromeric assembly.

(E) GABA concentration-response curves for α5β3-EM (black) and α5β3-WT (gray) expressed in *Xenopus* oocytes. Larger circles indicate mean normalized current ± standard error of the mean for 7 independent oocytes. Smaller circles show individual values from each oocyte. *Inset* indicates Hill coefficients (n_H) for the Boltzmann equation fitted for each sample.

(F) Example traces for α5β3-WT (left, gray) and α5β3-EM (right, black) in response to ~EC_{20} GABA, with a transient co-application of 10 µM $ZnCl_2$. *Insets* indicate current versus time scales, with the vertical bars showing 50 nA of current.

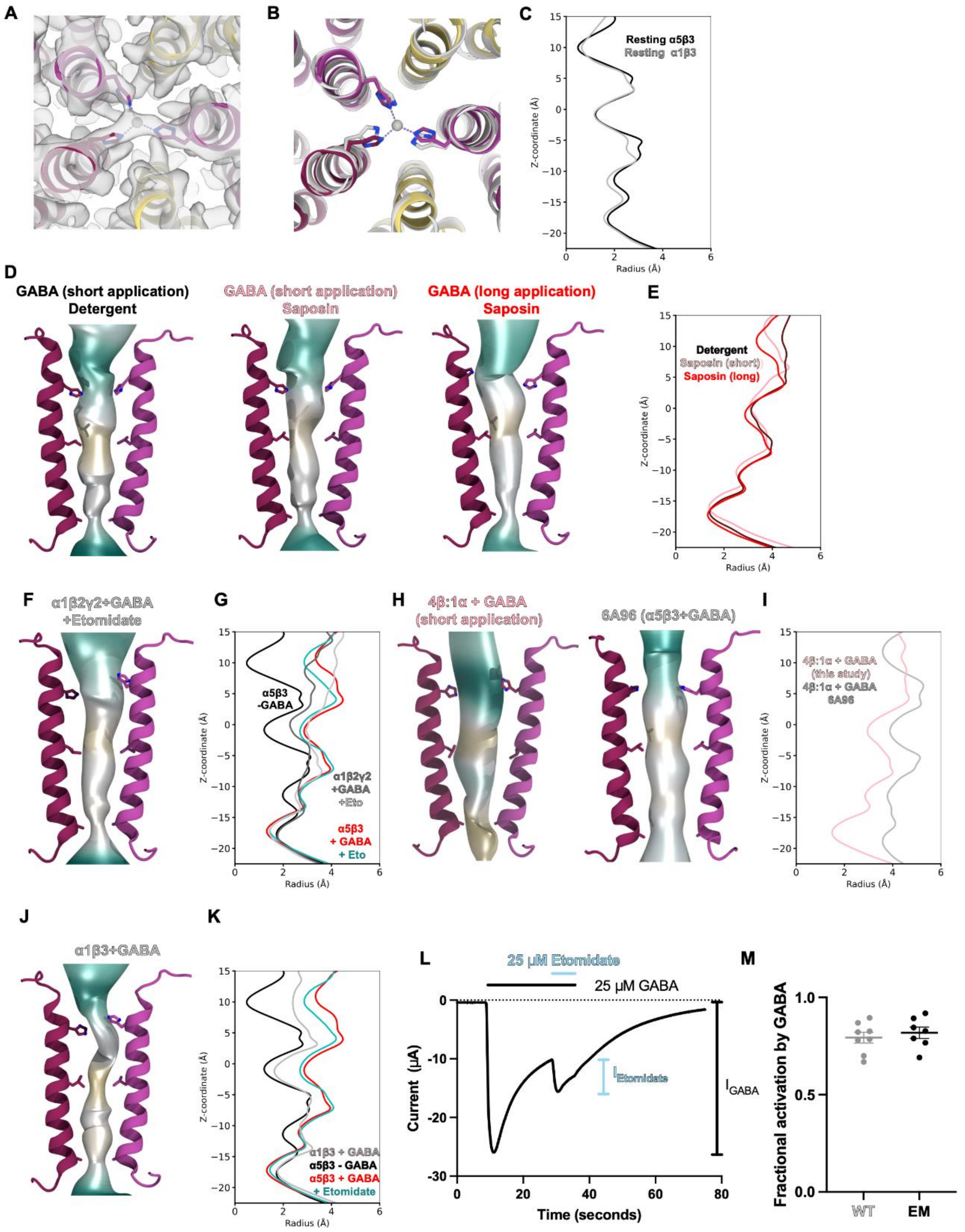

A
B
C
Resting α5β3
Resting α1β3
Z-coordinate (Å)
Radius (Å)
D
GABA (short application)
Detergent
GABA (short application)
Saposin
GABA (long application)
Saposin
E
Detergent
Saposin (short)
Saposin (long)
F
α1β2γ2+GABA
+Etomidate
G
α5β3
-GABA
α1β2γ2
+GABA
+Eto
α5β3
+ GABA
+ Eto
H
4β:1α + GABA
(short application)
6A96 (α5β3+GABA)
I
4β:1α + GABA
(this study)
4β:1α + GABA
6A96
J
α1β3+GABA
K
α1β3 + GABA
α5β3 - GABA
α5β3 + GABA
+ Etomidate
L
25 μM Etomidate
25 μM GABA
Current (μA)
Time (seconds)
I_Etomidate
I_GABA
M
Fractional activation by GABA
WT
EM

Supplementary Figure 5. Comparison of pore properties in α5β3-EM versus other $GABA_A$Rs.

(A) Density (gray surface) overlaid with model for the pore of resting-like α5β3-EM (PDB-9HAA), viewed from the extracellular side with M2-helices in α5 (yellow) and β3 subunits (magenta) as ribbons. Dashed lines indicate histidine coordination of a non-protein density in the pore, consistent with Zn^{2+}.

(B) Resting-like structure of α5β3-EM, viewed and colored as in A. Inhibitor-bound α1β3 (gray, PDB-7PC0)[28] is superimposed based on full alignment.

(C) Pore radius as a function of z-coordinate relative to L9' for resting-like α5β3-EM (black) and α1β3[28] (gray) structures.

(D) Permeation pathways, depicted as in C, in GABA-bound α5β3-EM, in detergent (left, PDB-9HNQ) or nanodiscs with short (center, PDB-9HUM) or long (right, PDB-9HNS) applications of GABA.

(E) Pore radius relative to L9' for GABA-bound α5β3-EM in detergent (black) or nanodiscs with short (pink) or long (red) GABA applications.

(F) Permeation pathways, depicted as in C, in GABA-bound structures with 1:4 α:β stoichiometry reported here (left, PDB-9HNR) or previously (right, PDB-6A96)[25].

(G) Pore radius relative to L9' for GABA-bound structures with 1:4 α:β stoichiometry reported here (pink) or previously (gray)[25].

(H) Permeation pathway, depicted as in C, in α1β2γ2 with GABA and etomidate bound (PDB-6X3V)[33].

(I) Pore radius relative to L9' for GABA-bound α5β3-EM in the presence (cyan, PDB-9HNT) or absence (red, PDB-9HUM) of etomidate, or α1β2γ2[33] in the presence (gray, PDB-6X3V) or absence (black, PDB-6X3Z) of etomidate.

(J) Permeation pathway, depicted as in C, in a GABA-bound α1β3 subtype (PDB-7PBD)[28].

(K) Pore radius relative to L9' for GABA-bound structures of α5β3-EM in the presence (cyan, PDB-9HNT) or absence (red, PDB-9HUM) of etomidate, or of α1β3 in the presence of GABA (gray, PDB-7PBD)[28].

(L) Example trace for α5β3-EM showing estimation of the fractional activation by GABA, defined as the ratio of I_{GABA} (black bar) to the sum of I_{GABA} and $I_{Etomidate}$ (blue bar).

(M) Fractional activation of α5β3-EM (black) and α5β3-WT (gray) by GABA. Bars indicate mean values ± standard error from 7 (α5β3-EM) or 8 (α5β3-WT) independent oocytes, also shown as points.

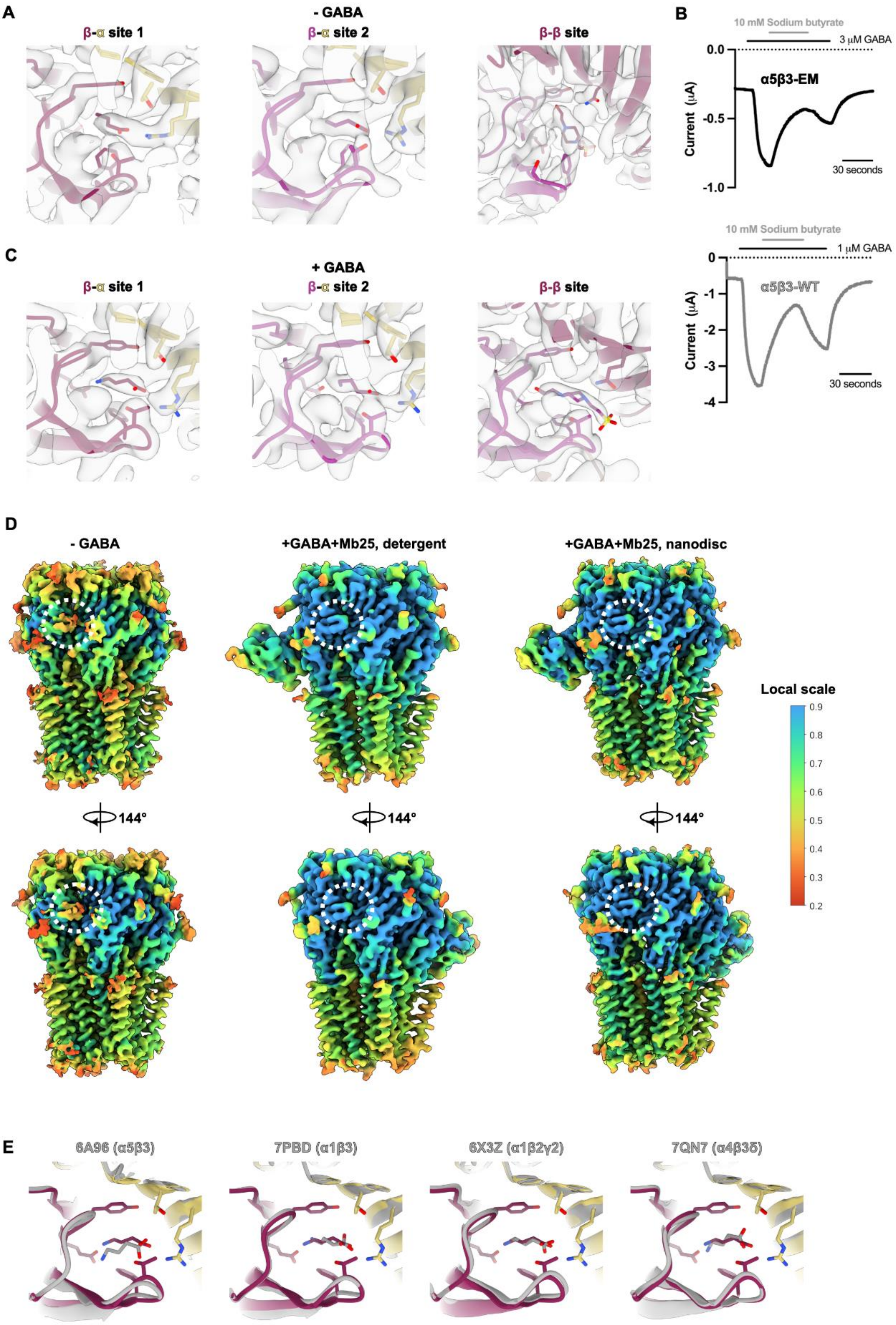
A
- GABA
β-α site 1
β-α site 2
β-β site
B
10 mM Sodium butyrate
3 μM GABA
α5β3-EM
Current (μA)
0.0
-0.5
-1.0
30 seconds
10 mM Sodium butyrate
1 μM GABA
α5β3-WT
Current (μA)
0
-1
-2
-3
-4
30 seconds
C
+ GABA
β-α site 1
β-α site 2
β-β site
D
- GABA
+GABA+Mb25, detergent
+GABA+Mb25, nanodisc
144°
144°
144°
Local scale
0.9
0.8
0.7
0.6
0.5
0.4
0.3
0.2
E
6A96 (α5β3)
7PBD (α1β3)
6X3Z (α1β2γ2)
7QN7 (α4β3δ)

Supplementary Figure 6. Comparison of extracellular ligand-binding pockets across α5β3-EM and related structures.

(A) Density map (gray surfaces) overlaid with atomic model at orthosteric site 1 (left), orthosteric site 2 (center) and an equivalent region of the β-β interface (right), all in resting-like α5β3-EM in micelles (PDB ID 9HAA). Each site is viewed from the extracellular side with α5 (yellow) and β3 subunits (magenta) as ribbons. Butyrate and HEPES (magenta), modeled at β-α and β-β interfaces respectively, along with coordinating side chains, are shown as sticks and colored by heteroatom.

(B) Example traces for α5β3-EM (top, black) and α5β3-WT (bottom, gray) in response to ~EC_{50} GABA, with a transient co-application of 10 mM sodium butyrate.

(C) Density map (gray surfaces) overlaid with atomic model at sites depicted and colored as in panel a, but in GABA-bound α5β3-EM in micelles (PDB ID 9HNQ). Ligands at β-α and β-β interfaces are assigned to GABA and HEPES, respectively.

(D) OccyPy analysis of density maps for resting-like α5β3-EM (left, PDB ID 9HAA), GABA-bound α5β3-EM in micelles (center, PDB ID 9HNQ) and GABA-bound α5β3-EM in nanodiscs (right, PDB ID 9HUM). Maps are colored according to local scale according to the scale bar at left. White dashed circles indicate orthosteric sites 1 (top) and 2 (bottom).

(E) Orthosteric site 1 in previously reported GABA-bound structures of (left to right, gray) α5β3 (PDB ID 6A96)[25], α1β3 (PDB ID 7PBD)[28], α1β2γ2 (PDB ID 6X3Z)[33] and α4β3δ $GABA_A$Rs (PDB ID 7QN7)[35], viewed as in panel a, with GABA shown as sticks. For comparison, GABA-bound α5β3-EM is superimposed with each structure based on the complementary α-subunit, and colored as in panel c, with GABA and coordinating side chains shown as sticks.

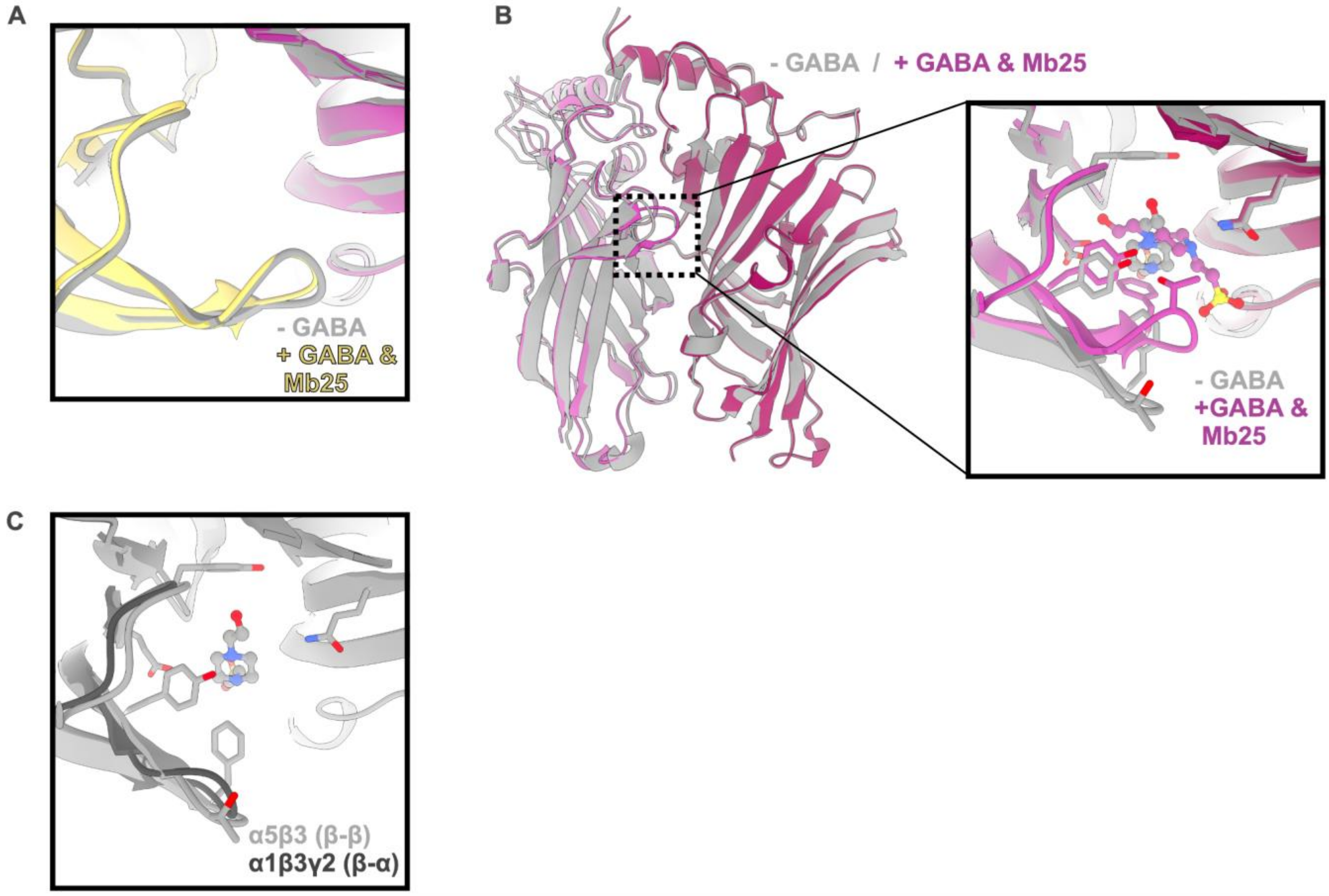

Supplementary Figure 7. Rearrangements at β-β and α-β ECD interfaces.

(A) An α-β ECD interface of GABA-bound α5β3-EM, viewed as in panel a-*inset*, showing α5 (yellow) and β3 subunits (magenta) as ribbons. For comparison, the resting-like state (gray) is superimposed based on the complementary β-subunits.

(B) The β-β ECD interface of GABA-bound α5β3-EM (magenta, PDB ID 9HNQ) superimposed with the resting-like state (gray, PDB ID 9HAA) based on the complementary-subunit ECD, viewed from the membrane plane. *Inset* shows a zoom of the boxed region, viewed with 90° rotation from the extracellular side, with HEPES molecules shown as balls-and-sticks and neighboring side chains as sticks, colored by heteroatom.

(C) The β-β ECD interface of resting-like α5β3-EM (gray), depicted as in panel a-*inset*, superimposed with orthosteric site 1 at the β-α interface of a PTX-bound α1β3γ2 $GABA_A$R (black, PDB ID 6HUG)[36] based on the complementary subunits.

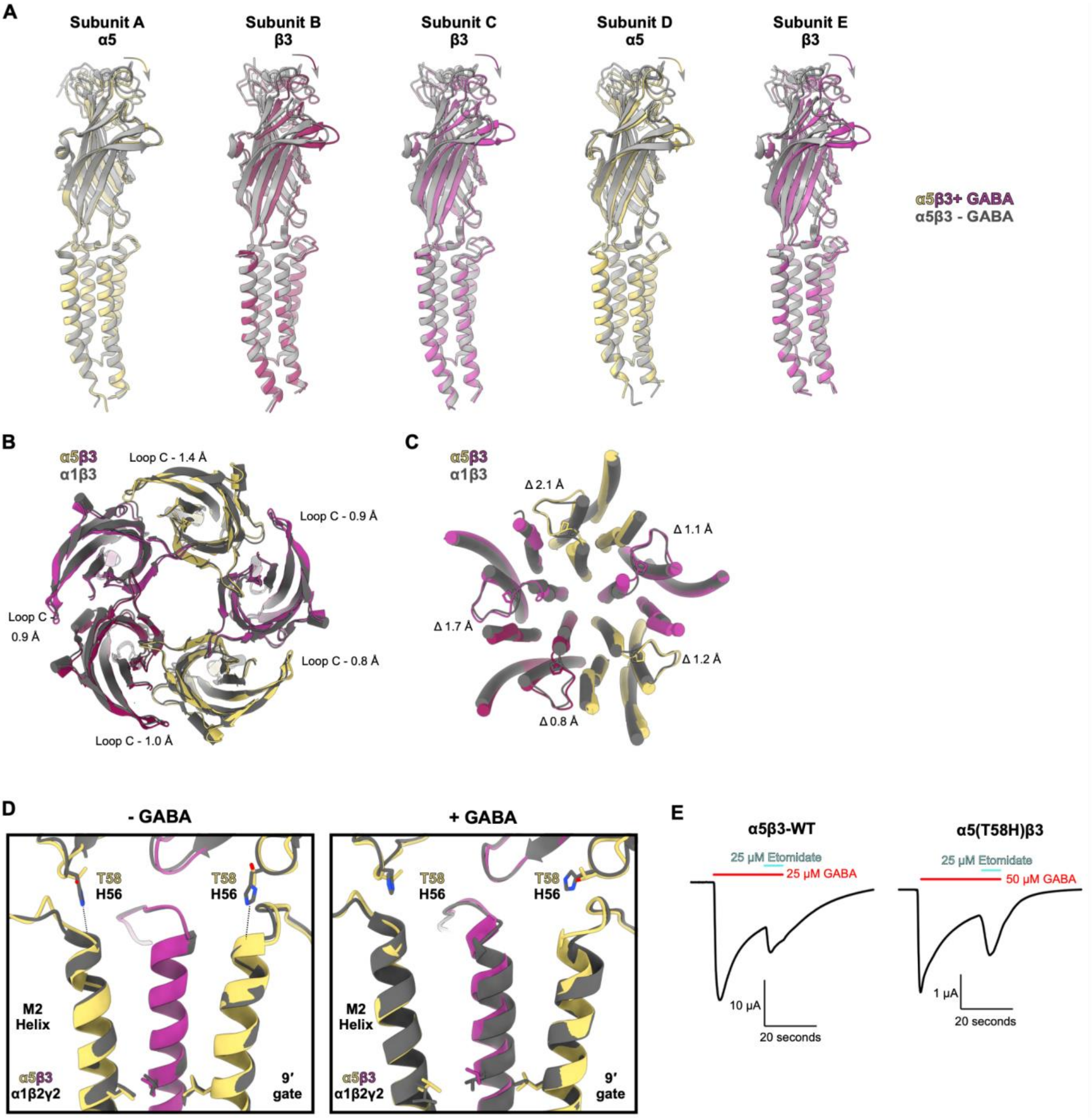

A
Subunit A
α5
Subunit B
β3
Subunit C
β3
Subunit D
α5
Subunit E
β3
α5β3+ GABA
α5β3 - GABA
B
α5β3
α1β3
Loop C - 1.4 Å
Loop C - 0.9 Å
Loop C
0.9 Å
Loop C - 0.8 Å
Loop C - 1.0 Å
C
α5β3
α1β3
Δ 2.1 Å
Δ 1.1 Å
Δ 1.7 Å
Δ 1.2 Å
Δ 0.8 Å
D
- GABA
+ GABA
T58
H56
M2
Helix
α5β3
α1β2γ2
9′
gate
E
α5β3-WT
α5(T58H)β3
25 μM Etomidate
25 μM GABA
50 μM GABA
10 μA
1 μA
20 seconds

Supplementary Figure 8. Comparisons between α5- and α1-containing $GABA_A$Rs.

(A) Five individual subunits of GABA-bound α5β3-EM (PDB ID 9HNQ), with α5 (yellow) and β3 (magenta). For comparison, the equivalent subunit in resting-like α5β3-EM (gray, PDB ID 9HAA) is superimposed based on the subunit TMD. Arrows indicate relative pivot of each subunit ECD relative to its TMD.

(B) Extracellular view of GABA-bound α5β3-EM (PDB ID 9HNQ), showing an ECD slice as in Fig. 4a, with α5 (yellow) and β3 (magenta) subunits labeled according to chain ID. For comparison, a GABA-bound α1β3-$GABA_A$R structure (gray, PDB ID 7PBD)[28] is superimposed based on the five TMD subunits. Distances labeled below loop C in each subunit indicate displacement of Cα atoms at the tip of each loop C (α1/5-T209 or β3-T202) between α5- and α1-containing structures.

(C) TMD slice as in Fig. 4c of GABA-bound α5β3-EM and α1β3 $GABA_A$R[28] structures, colored as in panel a. Distances labeled below the M2–M3 loop in each subunit indicate displacement of Cα atoms at the midpoint of each M2–M3 loop (α1/5-P281 or β3-P273) between α5- and α1-containing structures.

(D) Structures of α5β3-EM in the absence (left, PDB ID 9HAA) or presence (right, PDB ID 9HNQ) of GABA, depicted as in Fig. 4e. For comparison, structures of α1β2γ2 $GABA_A$Rs (gray) with bicuculline (left, PDB ID 6X3S)[33] or GABA+etomidate (right, PDB ID 6X3V)[33] are superimposed based on the highlighted regions. Residues at the midpoint of the β1–β2 loop and activation gate are shown as sticks and colored by heteroatom. Dashed lines indicate prospective hydrogen bonds between the side chain of α1-H56 and backbone of the M2-helix in the bicuculline-inhibited state.

(E) Example electrophysiology traces for α5β3-WT (left) and α5(T58H)β3 (right) in response to a saturating pulse of GABA (25 µM and 50 µM respectively) followed by addition of 25 µM etomidate and subsequent washout. *Insets* indicate current versus time scales.

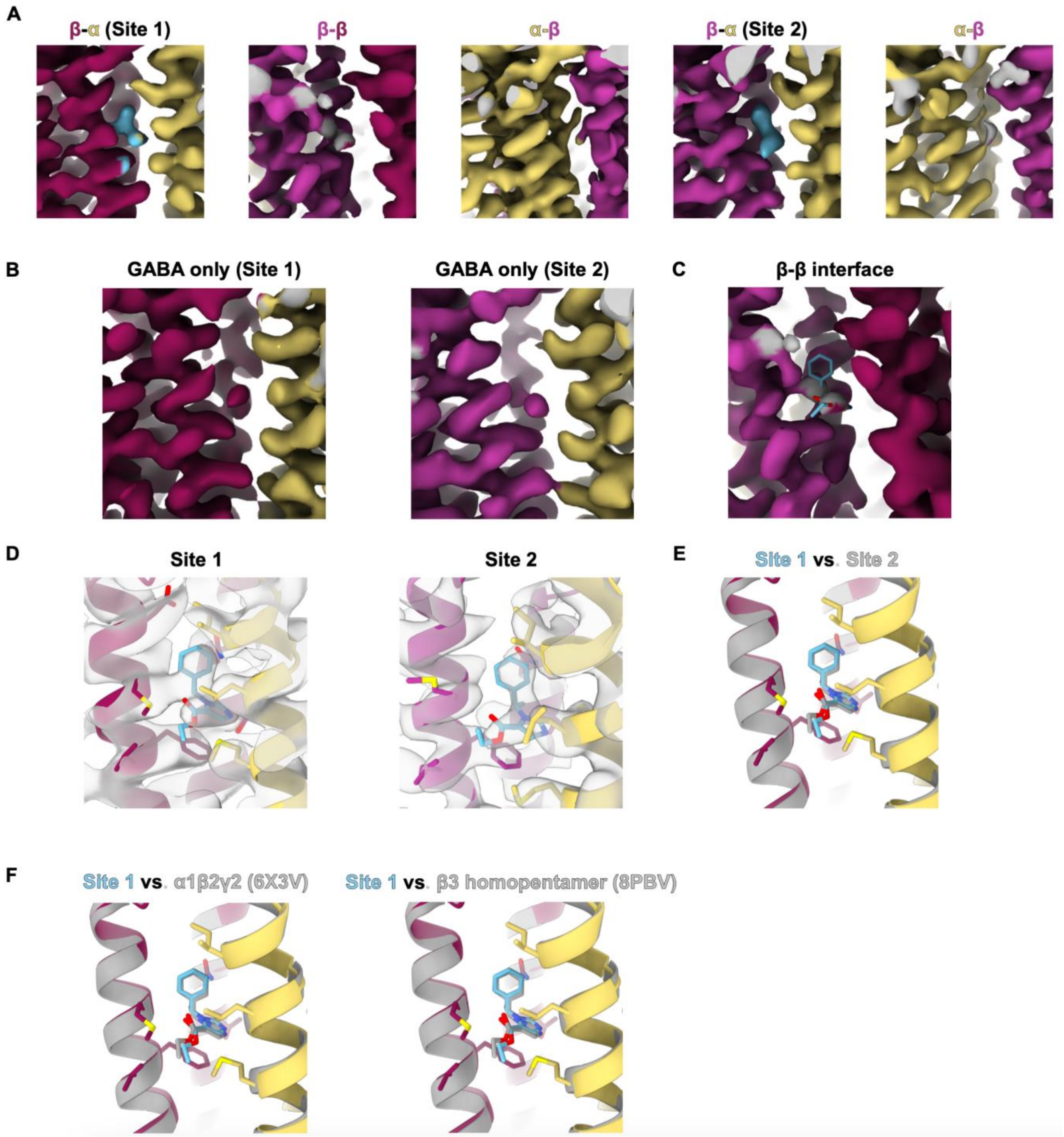

Supplementary Figure 9. Etomidate binding to α5β3 $GABA_A$Rs.

(A) Density map at five subunit interfaces in α5β3-EM bound to etomidate (PDB ID 9HNT), colored as in Fig. 5b.

(B) Density map at β-α interfaces in GABA-bound α5β3-EM (PDB ID 9HUM), depicted as in panel a.

(C) Density maps at the β-β interface in α5β3-EM bound to etomidate, depicted as in panel a. An overlaid etomidate molecule (cyan sticks) fits poorly to the available density, and could not be reliably built.

(D) Density map (gray surface) overlaid with the atomic model at β-α interfaces in α5β3-EM bound to etomidate, depicted as in panel a.

(E) Overlay between TMD sites 1 (colored) and 2 (gray) in α5β3-EM bound to etomidate, overlaid on the full receptor and depicted as in panel d.

(F) TMD site 1 in α5β3-EM bound to etomidate, depicted as in panel e. For comparison, etomidate-bound structures (gray) of α1β2γ2 (left, PDB ID 6X3V)[33] or β3-homopentamer (right, PDB ID 8PVB)[39] $GABA_A$Rs are superimposed based on the β-α interface, showing similar binding poses for etomidate.

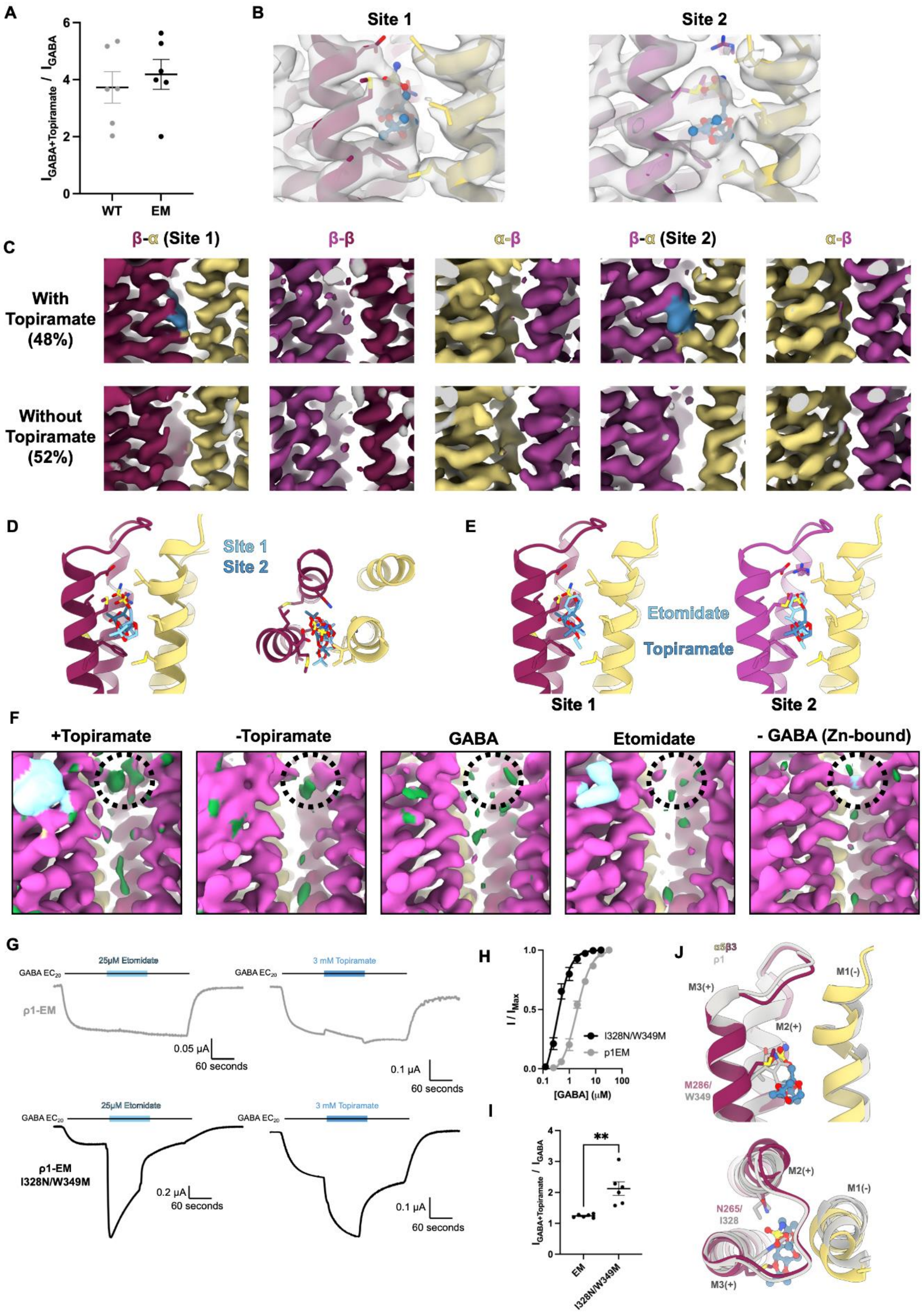

A
B
Site 1
Site 2
WT
EM
C
β-α (Site 1)
β-β
α-β
β-α (Site 2)
α-β
With Topiramate (48%)
Without Topiramate (52%)
D
Site 1
Site 2
E
Etomidate
Topiramate
Site 1
Site 2
F
+Topiramate
-Topiramate
GABA
Etomidate
- GABA (Zn-bound)
G
GABA EC20
25μM Etomidate
3 mM Topiramate
ρ1-EM
0.05 μA
60 seconds
0.1 μA
60 seconds
ρ1-EM I328N/W349M
0.2 μA
60 seconds
0.1 μA
60 seconds
H
I / IMax
[GABA] (μM)
I328N/W349M
p1EM
I
**
EM
I328N/W349M
J
α5β3
ρ1
M3(+)
M1(-)
M2(+)
M286/ W349
N265/ I328

Supplementary Figure 10. Topiramate binding to α5β3 $GABA_A$Rs pocket

(A) Enhancement of GABA-evoked current by topiramate for α5β3-WT (gray) and α5β3-EM (black) using the protocol in Fig. 5a. Bars indicate mean values ± standard error from six oocytes, also shown as points.

(B) Density (gray) overlaid with the atomic model at β-α interfaces in α5β3-EM bound to topiramate (PDB ID 9RL5), depicted as in Supplementary Fig. 9d.

(C) Density map at five subunit interfaces in α5β3-EM structures with (top, PDB-9RL5) and without (bottom, PDB ID 9RPB) topiramate, resolved from the same dataset, depicted as in Supplementary Fig. 9a.

(D) TMD-sites 1 (light blue) and 2 (dark blue) in α5β3-EM bound to topiramate, depicted as in Supplementary Fig. 9, viewed from the membrane plane (left) or from the extracellular side (right).

(E) Overlay between etomidate (cyan) and topiramate (blue) binding poses in TMD-sites 1 (left) and 2 (right), shown withα5β3-EM bound to topiramate, depicted as in panel d.

(F) Densities inside the pore of α5β3-EM structures determined (left to right) for Mb25+GABA+topiramate (PDB-9RL5); Mb25+GABA, topiramate not resolved (PDB-9RPB); Mb25+GABA (PDB-9HUM); Mb25+GABA+etomidate (bottom, PDB-9HNT); and in a resting-like state (PDB-9HAA). Non-protein density (green) is evident in the pore of structures determined with topiramate, in a similar position as Zn^{2+} (cyan) in the resting-like structure.

(G) Example traces showing insensitivity of ρ1-EM $GABA_A$Rs to 25 µM etomidate or 3 mM topiramate (top) at GABA EC_{20}. The double I328N/W349M mutant is potentiated by both drugs (bottom).

(H) GABA concentration-response curves for ρ1-EM (gray) and ρ1(I328N/W349M)-EM (black). Error bars indicate ± standard error from three independent oocytes.

(I) Enhancement of GABA-elicited current by coapplication with topiramate for ρ1-EM and ρ1(I328N/W349M)-EM using the protocol in G. Solid bars indicate mean values ± standard error of the mean from six independent oocytes, also shown as individual points. Asterisks denote significance with P=0.0025 in two sided t-test.

(J) TMD-site 1 in α5β3-EM bound to topiramate, depicted as in D and viewed from membrane plane (top) or extracellular side (bottom). An equivalent region of ρ1-EM bound to GABA (gray, PDB-8OP9)[43] is superimposed by the principal subunits.
