# Supplementary material for: Structural basis for activation and potentiation in a human α5β3 GABAA receptor
Source: Nat Commun. 2026 Jun 15;17:5289. doi: 10.1038/s41467-026-74279-3 (PMC13269941; doi:10.1038/s41467-026-74279-3)
Supplement: Supplementary file 3 — Transparent Peer Review File [file 41467_2026_74279_MOESM3_ESM.pdf]

nature portfolio

Peer Review File

Structural basis for activation and potentiation in a human α5β3 $GABA_A$ receptor

Corresponding Author: Dr Rebecca Howard

This manuscript has been previously reviewed at another journal. This document only contains information relating to versions considered at Nature Communications.

This file contains all reviewer reports in order by version, followed by all author rebuttals in order by version.

Version 0:

Reviewer comments:

Reviewer #1

(Remarks to the Author)
The authors have adequately addressed the reviewers' comments and critiques. The additional topiramate structure of alpha5beta3, and mutagenesis data of rho1 to confer topiramate and etomidate sensitivity further enhance the impact of the study.

Reviewer #2

(Remarks to the Author)
The article describes the structure of the alpha5beta3 GABAA receptor in the presence of ligands, including topiramate and etomidate, via cryo-electron microscopy. The study appears well executed and for the most part reliable. The number of GABAA receptor structures available make the work less significant than it could be, however the wealth of information is impressive, the paper well written and the work thorough.

I have two concerns. The first is the electrophysiological experiments. Etomidate was applied to oocytes that were exposed to GABA midway through the GABA exposure, according to the figures. Unless I am confused about how this experiment was done, this is not an appropriate way to estimate open probability. The reason for this is that desensitized receptors will be present at different ratios during the decay phase of the trace. Instead, maximum GABA should be applied and allowed to wash out, then maximum GABA with etomidate should be applied. The method used cannot compare different receptor subtypes (or mutant receptors for that matter, if that is what you are doing). The curve fitting also needs more clarification, it would be conventional to fit to a Hill equation?

The second is topiramate. The concentrations that topiramate has shown to modulate GABAA receptors is high, and even with saturating concentrations there is not fully bound receptors in the protein preparation. Would the millimolar concentrations used be higher than the therapeutic concentrations and are more akin to those in overdose? While I appreciate that care was written in the manuscript not to overstate the results, it should be more definitively stated whether the concentrations of topiramate that modulate the receptors are in the therapeutic range or not. While senior researchers are likely to check this for themselves, it is better in my experience to be clear on this point to less experienced researchers. After all, it is important to know not only drug mechanisms at therapeutic concentrations but also mechanisms at the concentrations it has adverse effects.

Version 1:

Reviewer comments:

Reviewer #2

(Remarks to the Author)
This is a revision of a manuscript that I have previously reviewed.

The authors have responded to my comments and made appropriate clarifications. I have nothing more to add.

Open Access This Peer Review File is licensed under a Creative Commons Attribution 4.0 International License, which permits use, sharing, adaptation, distribution and reproduction in any medium or format, as long as you give appropriate credit to the original author(s) and the source, provide a link to the Creative Commons license, and indicate if changes were made.
In cases where reviewers are anonymous, credit should be given to 'Anonymous Referee' and the source.
The images or other third party material in this Peer Review File are included in the article's Creative Commons license, unless indicated otherwise in a credit line to the material. If material is not included in the article's Creative Commons license and your intended use is not permitted by statutory regulation or exceeds the permitted use, you will need to obtain permission directly from the copyright holder.
To view a copy of this license, visit https://creativecommons.org/licenses/by/4.0/

Response to Reviewers: NCOMMS-25-85294-T

In the rebuttal below, reviewer comments are black, and responses are in blue.

Reviewer #1 (Remarks to the Author): The authors have adequately addressed the reviewers' comments and critiques. The additional topiramate structure of alpha5beta3, and mutagenesis data of rho1 to confer topiramate and etomidate sensitivity further enhance the impact of the study.

We thank the reviewer for their constructive comments and suggestions during the review process.

Reviewer #2 (Remarks to the Author): The article describes the structure of the alpha5beta3 GABAA receptor in the presence of ligands, including topiramate and etomidate, via cryo-electron microscopy. The study appears well executed and for the most part reliable. The number of GABAA receptor structures available make the work less significant than it could be, however the wealth of information is impressive, the paper well written and the work thorough. *Comment #2.1:* I have two concerns. The first is the electrophysiological experiments. Etomidate was applied to oocytes that were exposed to GABA midway through the GABA exposure, according to the figures. Unless I am confused about how this experiment was done, this is not an appropriate way to estimate open probability. The reason for this is that desensitized receptors will be present at different ratios during the decay phase of the trace. Instead, maximum GABA should be applied and allowed to wash out, then maximum GABA with etomidate should be applied. The method used cannot compare different receptor subtypes (or mutant receptors for that matter, if that is what you are doing).

We regret the imprecision of our original revision in this regard, and appreciate the opportunity to clarify. Although we originally used the term "efficacy," the reviewer rightly points out that we were not actually assessing maximum open probability, as implied by this term. Efficacy is generally defined as the proportion of receptors reaching the open state as a fraction of the total available receptor pool, and can be assessed as described by the reviewer. We were rather interested in measuring the fraction of receptors reaching either the open or desensitized states as a fraction of the total available pool, which we now define as "activation propensity." We have now rephrased all mentions of receptor efficacy to focus instead on activation propensity, and added a detailed explanation of our experimental-analytical protocol and rationale in **Methods**:

"To assess the extent of potential receptor activation with saturating GABA, we estimated the proportion in open and desensitized states as a fraction of the total receptor pool, defined here as the GABA activation propensity (Extended Data 5l). We adopted this metric as a variation on conventional measures of efficacy, i.e. the ability of a ligand to drive channels into the open state, given that we were unable to directly observe the open state in our cryo-EM datasets. To quantify activation propensity, we chose an electrophysiological protocol motivated by steady-state inactivation (h_∞) measurements[57]. An initial pulse of saturating GABA was presumed to produce a variety of GABA-bound states including resting-like/primed (pre-active)

and open/desensitized (activated); further addition of etomidate, with continued saturating GABA, was presumed to drive all pre-active receptors into activated states. We also assumed that the number of receptors driven into activated states with a pulse of either GABA or GABA+etomidate was proportional to the change in current amplitude from the moment before the pulse to the peak during the pulse. Thus, to estimate the fraction of channels driven into activated states with GABA alone, we divided the current change elicited with GABA (I_{GABA}) by the sum of the current changes induced with GABA and GABA+etomidate (I_{GABA} + $I_{Etomidate}$, Extended Data Figure 5I). Since this analysis lumps together the open and desensitized states, it should not be heavily influenced by differences in the extent of desensitization between channel variants. It should also minimize confounds from kinetic effects of etomidate on rates of opening or desensitization[58,58], which can overestimate the change in peak current between isolated GABA and GABA+etomidate pulses. Notably, this protocol approximates the fraction of channels still available for activation seconds after the application of GABA, which roughly corresponds to our timepoint of cryo-EM grid freezing."

Comment #2.2: The curve fitting also needs more clarification, it would be conventional to fit to a Hill equation?

Thank you for catching this; we did indeed use the Hill equation rather than the Boltzmann equation for curve fitting. We have corrected the error, and added the formulation of the equation used for fitting the curve to **Methods**:

"To quantify GABA activation, concentration-response curves were fitted by nonlinear regression to the Hill equation below with variable slope using Prism 9.4 (GraphPad Software):

$$I/IMax \ = \frac{[GABA]^n}{[GABA]^n + {EC_{50}}^n}$$

where n is the Hill slope and EC_{50} is the concentration of half-maximal activation."

Comment #2.3: The second is topiramate. The concentrations that topiramate has shown to modulate GABAA receptors is high, and even with saturating concentrations there is not fully bound receptors in the protein preparation. Would the millimolar concentrations used be higher than the therapeutic concentrations and are more akin to those in overdose? While I appreciate that care was written in the manuscript not to overstate the results, it should be more definitively stated whether the concentrations of topiramate that modulate the receptors are in the therapeutic range or not. While senior researchers are likely to check this for themselves, it is better in my experience to be clear on this point to less experienced researchers. After all, it is important to know not only drug mechanisms at therapeutic concentrations but also mechanisms at the concentrations it has adverse effects.

We agree that the lack of saturated drug binding at millimolar concentrations of topiramate makes it unlikely that modulation of α5β3 $GABA_A$ receptors is therapeutically relevant, except in cases of overdose or in individuals with mutations in α5 or β3 which alter gating properties. We have added this point to the **Discussion**:

“Given that the millimolar concentrations of topiramate used in our cryoEM studies are insufficient to saturate the binding sites in our structures, modulation of α5β3 receptors by topiramate is unlikely at therapeutic doses (<85 μM plasma concentration), but may be relevant in case of overdose or in carriers of mutations in α5 or β3 with altered properties.[52]”
